# Developing and validating a university needs instrument to measure the psychosocial needs of university students

**DOI:** 10.1111/bjep.12515

**Published:** 2022-05-19

**Authors:** Richard Tindle, Paola Castillo, Natalie Doring, Leigh Grant, Royce Willis

**Affiliations:** ^1^ School of Health and Behavioural Sciences University of the Sunshine Coast Gympie QLD Australia; ^2^ Faculty of Business, Justice and Behavioural Sciences, School of Psychology Charles Sturt University Port Macquarie NSW Australia; ^3^ Kirby Institute University of New South Wales Sydney NSW Australia; ^4^ Faculty of Health Southern Cross University Coffs Harbour NSW Australia

**Keywords:** college students, higher education, psychological distress, psychosocial needs, university students

## Abstract

**Background:**

University students are four times more likely to experience elevated levels of psychological distress compared to their peers. The psychosocial needs of university students are associated with high psychological distress, stressful life events, and academic performance. Our study focuses on developing a measure to help universities identify these psychosocial needs.

**Aims:**

The study aimed to develop and validate the factor structure of the University Needs Instrument and identify the relationship between psychosocial needs, psychological distress and academic performance among university students.

**Sample:**

Undergraduate university students (*N* = 511) currently studying at university.

**Method:**

Participants completed demographic questions, the University Needs Instrument and the Kessler‐10 Psychological Distress scale. The University Needs Instrument comprises 30 items within six psychosocial factors (academic support, financial support, support from family, support from friends, practical support and emotional support), each consisting of five items.

**Results:**

Confirmatory factor analysis showed that all items significantly loaded on the six hypothesized factors. The hypothesized model was supported by the data displaying excellent model fit and psychometric properties. Our analysis determined that the UNI has strong internal consistency. The results also confirmed that university students' high levels of psychological distress and their academic performance may be affected by their psychosocial needs.

**Conclusions:**

Our findings have provided an initial validation of the UNI to help screen and identify the psychosocial needs of university students. This scale can be used to identify the appropriate psychosocial support that can be offered to students and in turn could help reduce their psychological distress, improve their psychosocial well‐being and increase academic performance.

## INTRODUCTION

University students are four times more likely to experience elevated levels of psychological distress compared to their non‐university student peers of the same age (Leahy et al., 2010; Sharp & Theiler, 2018). Student's increased levels of psychological distress can lead to a reduction in academic performance (Sharp & Theiler, 2018), the development of unhealthy behaviours (e.g., poor diet, reduced exercise and irregular sleeping patterns; (Lund et al., 2010; Robotham & Julian, 2006), and an increased risk of developing mental health problems (Stallman & Shochet, 2009). Other psychosocial factors associated with psychological distress in university students include socio‐economic, socio‐demographic and academic factors (Sharp & Theiler, 2018). There is a need for universities to improve support services for undergraduates (McKenzie & Schweitzer, 2001; Sharp & Theiler, 2018; Wibrowski et al., 2017) to reduce their levels of psychological distress, enhance their university experience and reduce attrition from their study (McGaha & Fitzpatrick, 2005). Our study aims to identify the psychosocial needs of university undergraduate students and develop and identify the factor structure of the University Needs Instrument (UNI).

### Student engagement surveys

The National Survey of Student Engagement (NSSE; Kuh, 2001) is one of the most widely used questionnaires of student engagement in the USA and Canada with similar adaptions made for Australian and New Zealand students (e.g., Australasian Survey of Student Engagement; Coates, 2010), and for students within the United Kingdom (e.g., UK Engagement Survey; Buckley, 2014; Neves, 2019). All three instruments are used by institutions in their relevant countries to identify, benchmark, and compare undergraduate students' engagement, skills development, time spent studying, academic integration, and learning outcomes. The student engagement surveys have identified several areas associated with poor student engagement that could be contributing to attrition. For instance, the top reasons for why students leave universities were health‐related (family or self), financial work‐life balance difficulties, incorrect choice of degree, not enough support for their learning (i.e., from academic staff) or not enough support for personal issues (Coates, 2010; Neves, 2019).

The national engagement surveys have provided critical insights into student engagement, retention and completion. However, the research does not consider the impact of psychological well‐being on students during their studies. Although there are high completion rates among undergraduate students, the requirements needed to complete their studies often contribute to high levels of stress. Further understanding of the types of psychosocial needs associated with academic, financial, social and emotional support will help provide universities with resources to not only reduce attrition but improve the university student experience and the psychological well‐being of their undergraduate students.

#### Tinto's model of academic persistence

Tinto's model of academic persistence (Tinto, 1993, 2012) identifies that students' decisions to remain at university are dependent on how well they integrate academically and socially. Much of the emphasis on student retention is placed on the institution's role in the education process (Lee & Matusovich, 2016; Salami, 2011). For example, providing students with the appropriate support to meet academic demands (Barnett & Stamm, 2010; Clutterbuck et al., 2017; Parnes et al., 2020) and the opportunity to integrate socially with peers, staff and the institution (Berger & Braxton, 1998; Sidelinger et al., 2016). When students have a positive interaction and integration, where they can meet the academic and social demands of the institution (e.g., be provided with resources), they are more likely to remain at university. However, attrition becomes a risk factor when students have not successfully integrated academically or socially (Tinto, 2012).

One of the limitations of Tinto's model is the narrow focus on academic and social integration (Salami, 2011). Indeed, much of the research has focussed on isolating the effects of social integration (Berger & Braxton, 1998; Sidelinger et al., 2016) or academic integration (Barnett & Stamm, 2010; Clutterbuck et al., 2017; Parnes et al., 2020). In turn, there is still a limited understanding of other psychological and social factors that contribute to the university student experience. By identifying specific psychosocial factors of university students, institutions can provide more appropriate and targeted services to ensure students are supported psychologically and socially. Tinto's model provides an important starting point by identifying students' academic and social needs as predictors of retention and psychological well‐being. Further identifying the types of academic and social support, along with other psychosocial factors, will help identify the unique psychosocial needs of university students.

#### University student needs

Psychosocial needs refer to the psychological and social needs that contribute to an individual's psychological well‐being. In the context of university students, we define psychosocial needs as their desire or requirement to receive help or support for their psychological, social, and emotional well‐being. University students experience a range of psychosocial needs, relating to academic support (Wibrowski et al., 2017; Wilcox et al., 2005), financial support (Robbins et al., 2004; Walsh et al., 2010), emotional support (Beiter et al., 2015), support from family and friends (Çivitci, 2015; Wilcox et al., 2005), and practical support (Beiter et al., 2015; Lizzio et al., 2010; Walsh et al., 2010). Previous research has found that psychosocial factors among undergraduate university students are associated with psychological distress (Leahy et al., 2010; Sharp & Theiler, 2018). Wilcox et al. (2005) interviewed students to investigate their experiences as first‐year undergraduate students. Their study identified three major themes that were challenges for undergraduates, the need to develop social support from friends, family and colleagues; the need for academic support and development of independent learning; and more practical help relating to finances and travel to university. Wilcox et al.’s (2005) qualitative assessment suggested that university students have specific needs but providing resources aimed at improving opportunities for them to form interpersonal relationships is essential for undergraduates to acquire emotional support.

##### Academic support

Academic support is an important factor not only for how well undergraduates perform at university but also for their psychological well‐being (McKenzie & Schweitzer, 2001; Wibrowski et al., 2017). Further, Tinto's (1993) model of academic persistence suggests that one of the factors contributing to student retention is whether students can meet the academic demands of the institution. Other than predicting retention, personal stressors (e.g., family issues and loneliness) and academic stressors (e.g., the amount of material to learn and intellectual demands) significantly predicted stress (i.e., psychological morbidity on the General Health Questionnaire) in undergraduate students. Providing avenues to improve academic support can help increase psychological well‐being, reduce stress and improve retention (Edwards et al., 2016; Richardson, 2011; Walsh et al., 2010). Therefore, providing academic support resources (e.g., tutoring and mentoring programs, librarian assistance and study skills training) can foster academic success and retention (Bean & Eaton, 2001; Grillo & Leist, 2013; Hagel et al., 2012). Indeed, addressing students' academic needs can result in a reduction in attrition (Edwards et al., 2016) and an increase in learning (Richardson, 2011).

Another important aspect of academic support is the relationship between students and academics. Satisfaction with the student‐academic relationship is related to both academic performance, life satisfaction, and dispositional traits (Krumrei‐Mancuso et al., 2013; Retna et al., 2009). While preferred traits vary as a function of students' personality, overall students report greater satisfaction with the relationship when they perceive academics to be conscientious, open, approachable and stable (Furnham & Chamorro‐Premuzic, 2005). Students' satisfaction with this relationship is associated with continued engagement in education (Noble & Henderson, 2011). Yet only 37% of students in the UK reported being engaged with academic staff (Neves, 2019; Radloff & Coates, 2009). In Australia, 50% of students who seriously considered leaving higher education cited a lack of academic support as a reason for departure (Challice et al., 2021). These results emphasize the importance of students' relationships with academics. However, engagement with academic staff seems to be a poor performing area across the UK and Australian institutions (Neves, 2019; Radloff & Coates, 2009). Good relationships with teaching staff seem to enhance students' perception of academic support. When students feel supported by academic staff, they are more likely to be engaged with learning, and their perception of support is dependent on whether they have good relationships with the teaching staff.

##### Financial support

Universities Australia (2018) released a report identifying the current financial situation of 18,584 university students nationwide. In 2017, the median annual income for university students was $18,300, indicating that many students were living below the poverty line, which was about $22,500. The costs of attending university can be a source of stress for many students who need to pay for food, accommodation and university‐related resources such as textbooks, computers and transport. For example, the median annual cost associated with study‐related expenses for an undergraduate student is ~$1300 on top of their living expenses (~$12,300 annually). Of further concern, was that about 30% of students indicated that their estimated expenses were greater than their expected income. As such, it is unsurprising that most students report being worried about their finances (58%), with financial stress further exacerbated for regional students (64%) and Indigenous students (72%; Universities Australia, 2018). The burden of finances as an undergraduate student has been linked to higher levels of stress (Farrer et al., 2016; Walsh et al., 2010); and should be considered a priority area of need for undergraduates.

##### Support from family and friends

Social support from family and friends is essential for reducing stress in undergraduate students. For example, Chu‐Lien Chao (2012) identified that social support (i.e., friends, family, colleagues, and partners) and positive coping strategies were predictive of psychological well‐being. This study found that when students were able to access social support, they typically reported lower levels of stress. Further, the study noted an interaction between dysfunctional coping and a lack of social support. This indicates that when students were unhappy with the social support they received, they reported higher levels of stress. Similar findings have also suggested that forming social support with university peers is a strong predictor of positive psychological well‐being and reduced levels of distress (Çivitci, 2015; Robbins et al., 2004). For mature age students, family responsibilities such as children, housework and employment can hinder their ability to integrate into university and reduce the social support they receive (Stallman, 2008; Steele et al., 2005; van Rhijn et al., 2016; Wilcox et al., 2005). Currently, there are recommendations for universities to introduce or improve policies to create social support networks to support undergraduates' integration into university (van Rhijn et al., 2016). However, Robotham and Julian (2006) conducted a review of stressors among university/college students and found that, in addition to academic and financial concerns, one of the most commonly reported stressors for students was parental pressure and the need to perform well (Beiter et al., 2015). While social support typically improves psychological well‐being among undergraduates, when that support is perceived as unhelpful or causing additional pressure, it may contribute to higher levels of distress.

##### Practical support

The transition to university is stressful because students often relocate and this is usually their first time living out of home, without their parents, siblings and friends. During this time, students might require additional support to navigate the practical requirements of attending universities such as self‐management and scheduling. The practical requirements of attending university include managing study loads, timetabling, enrolling in classes and arranging transport to and from university (Beiter et al., 2015; Chu‐Lien Chao, 2012; Çivitci, 2015; Deatherage et al., 2014; Lizzio et al., 2010; Lund et al., 2010; Robotham & Julian, 2006; Stewart et al., 1997; Walsh et al., 2010). For many, it might be the first time they have been responsible for managing their schedules and they must do this while adapting to a new environment and the other demands of university.

Being able to cope and manage the practical requirements of attending university is an important determinant for student retention and can contribute to high levels of distress when students are unable to cope efficiently (Fedorková et al., 2020). Indeed, Omar et al., (2020) found that within a sample of dental students (*N* = 380), 64.8% reported that their study load was the highest source of stress. Their findings have confirmed recent developments demonstrating a negative association between study load and academic performance (Salih et al., 2021). These findings also support the notion that learning to manage new stressors at university (i.e., managing study load, timetables and enrolments) might preoccupy students and detract from their ability to focus on their academic performance (Harris‐Reeves & Mahoney, 2017). However, evidence suggests that these types of stressors might reduce as the students’ progress throughout their degrees. For example, Harris‐Reeves and Maloney (2017) showed that by the second semester, most first‐year students had developed the necessary coping skills and abilities to adequately navigate and manage the study demands of university. Nonetheless, while most students develop these skills, those that have not successfully adapted might need additional support to help cope with and develop the skills required to succeed at university.

Sleep is a practical need that has been extensively studied within university student populations (Gaultney, 2010; Gomes et al., 2011; Kabrita & Hajjar‐Muça, 2016; Lawson et al., 2019; Lemma et al., 2012; Lemma et al., 2014; Peltzer & Pengpid, 2015; Schlarb et al., 2017). Sleep deprivation and poor‐quality sleep are prevalent among university students and can contribute to poorer academic outcomes and higher levels of stress (Gomes et al., 2011; Lemma et al., 2012; Orzech et al., 2011). Not getting enough sleep, is associated with less engagement in class, poor daily functioning and is associated with lower assessment grades. Poor sleep quality is often exacerbated during periods of increased stress, such as exam period but often improves after exams and periods of heavy study load are completed (Campbell et al., 2018). However, much of this research has focussed on objective measures of sleep (e.g., sleep duration and deprivation) but there is emerging evidence of the distinction between objective and subjective measures of sleep.

Subjective sleep quality refers to an individual's psychological appraisal of their quality of sleep. For example, Ban and Lee (2001) found that even when university students had sufficient sleep duration (i.e., objective), more than 30% of the sample reported having insufficient sleep (subjective). Further, subjective sleep quality has been associated with perceptions of quality of life, physical health and psychological health (Carpi et al., 2022; Putilov et al., 2021; Simor et al., 2015). Indeed, studies have identified a relationship between university students' subjective sleep quality and their emotional states (Ahammed et al., 2021; Arbinaga et al., 2018; Simor et al., 2015; Norbury & Evans, 2019). These results have confirmed that university students who report poor subjective sleep quality also described having an increase in negative emotional affect (e.g., anxiety, anger and a reduction in positive emotional affect (e.g., enthusiastic, active and alert) during the day (Simor et al., 2015). These findings suggest, that while sleep duration can have significant implications for university students' academic performance and mental health; subjective perceptions of their quality of sleep also contribute to their psychological, physical and social well‐being. Taken together, the practical needs of students are important considerations as they affect their daily functioning, academic performance and contribute to higher levels of stress.

##### Support for emotional distress

Feelings of anxiousness, loneliness, frustration and depression are commonly reported among undergraduate students (Andrews & Wilding, 2004; Beiter et al., 2015; Eisenberg et al., 2007; Sharp & Theiler, 2018). Stressful situations often precipitate these emotions (e.g., assessment deadlines, financial burdens and exams) and undergraduates who are unable to cope with negative emotions effectively, typically report higher levels of psychological distress and poorer academic performance (Andrews & Wilding, 2004; Austin et al., 2010). Identifying undergraduates who need help coping with emotions is vital to improving their academic performance and reducing their levels of psychological distress.

There is a dynamic relationship between psychological health, academic success and study retention. Andrews and Wilding (2004) reported that by halfway through a semester, 20% of previously symptom‐free undergraduates met the criteria for anxiety, and 9% met the criteria for depression. Depression, anxiety and stress have been reported to predict decreased exam performance and increased attrition rates (Andrews & Wilding, 2004; Saunders‐Scott et al., 2018). Poor academic performance and attrition can have long‐term negative impacts on emotional well‐being (Smith, 1982). In other words, for some students, participation in study negatively impacts their well‐being, leading to poor outcomes, which potentially contributes to longstanding emotional distress. These findings highlight the need to develop a measure, like the University Needs Instrument, which can identify students at risk of developing emotional distress.

#### Identifying the needs of university students

**FIGURE 1:**
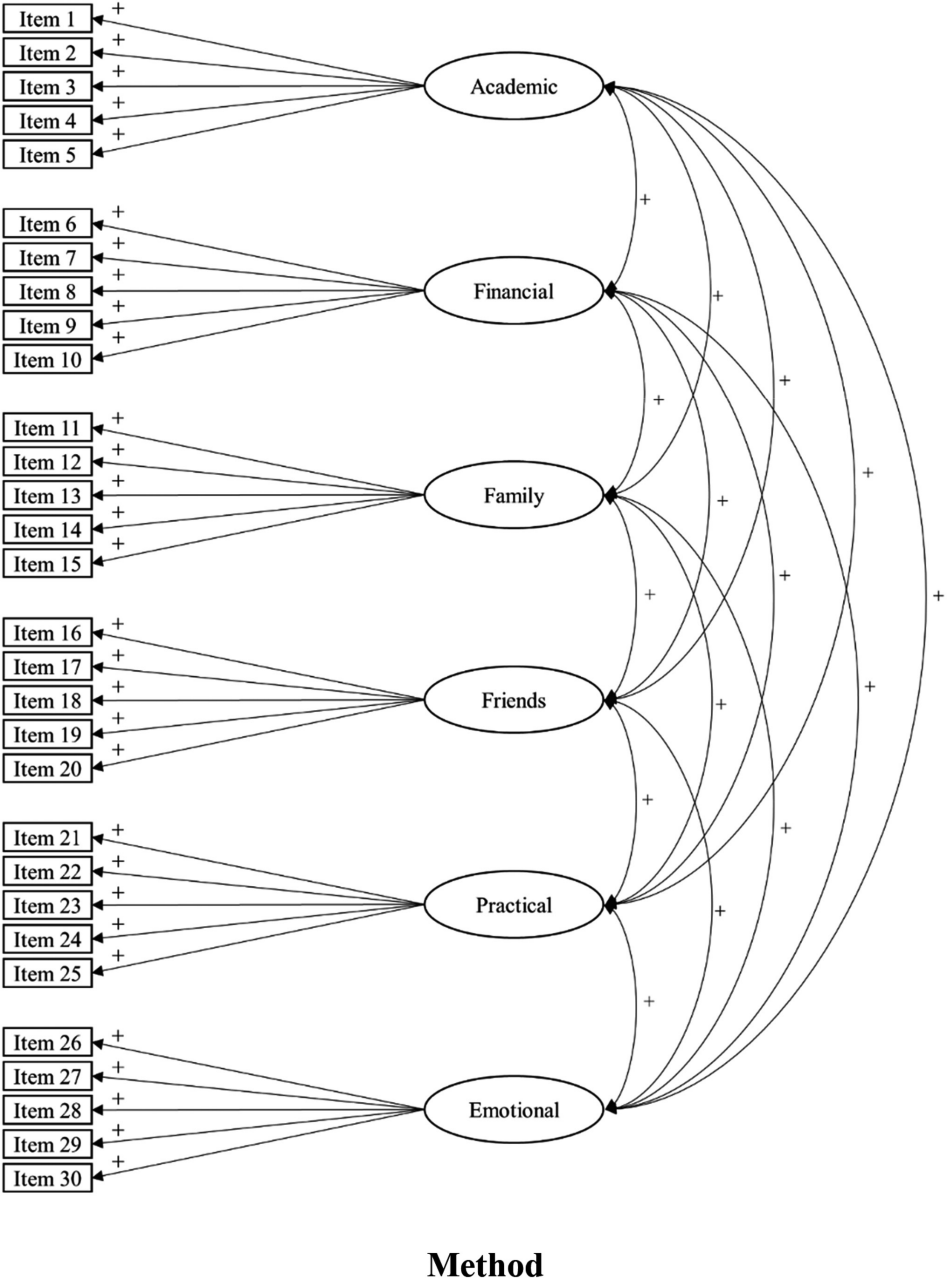
The hypothesized factor structure for the university needs instrument. The ‘+’ signs indicated the predicted association between each item and their latent factor and the predicted direction for the covariance between factors

The importance of identifying undergraduate students who are experiencing heightened levels of psychological distress has been demonstrated consistently by recent research. However, little of this research has focused on developing a measure that would help universities identify these psychosocial needs. While psychosocial needs may not be the precipitating causal factor for psychological distress, student perceptions of their learning environment are the strongest predictor of learning outcomes (Lizzio et al., 2010). By identifying the psychosocial needs of undergraduate students, universities can develop appropriate resources and provide support services to improve the students' perceptions and experience of studying at university. However, it would be uneconomical for universities to exhaustively investigate every possible psychosocial need of their students. Accordingly, a scale identifying the most prominent needs of university students is desirable. Based on the literature (reviewed above) the prominent psychosocial needs of university students were identified as academic needs, financial needs, family support needs, friend support needs, practical needs, and emotional support needs. In our study, we use confirmatory factor analysis to identify the factor structure of the University Needs Instrument to measure the psychosocial needs of undergraduate university students. The hypothesized factor structure, factor loadings, and covariance are presented in Figure 1. However, as this is the initial validation of the individual factors we did not aim to identify if the latent factors comprised a higher‐order factor. Further, convergent validity was assessed by assessing the correlation coefficients between individual items (and their factors) and psychological distress and students' GPA.

## METHOD

### Participants

The minimum sample size for the study was determined based on previous research which conducted Monte‐Carlo simulations to determine optimal sample sizes for conducting confirmatory factor analysis (see Bujang et al., 2018). The recommendation from these studies was a variable ratio of 1:2, 1:3, 1:5, and 1:10 (Bujang et al., 2018). However, based on the recommendations of these studies for using continuous variables (Bujang et al., 2018), a minimum ratio of 1:10 was used, translating to a minimum sample of 300 participants.

In total, 511 undergraduate students completed the survey. Most participants were from the University of the Sunshine Coast (*n* = 386; 75.5%), the remaining participants were enrolled at 30 different universities within Australia (*n* = 107; 20.94%), abroad (*n* = 9; 1.76%) or the university was not provided (*n* = 12; 2.35%). Participants were studying across 46 different degrees. However, the largest proportion of participants were studying psychology (*n* = 258; 50.49%), occupational therapy (*n* = 57; 11.15%), social science (*n* = 22; 4.31%), and only 14 (2.74%) participants did not indicate a degree.

Table 1 presents the demographic variables of the participants. Participants were aged between 17 and 35 (*M*_age_ *=* 27.82, *SD*_age_ = 11.69), studied between 0 and 76 h per week (*M =* 27.82*, SD* = 11.53), and worked between 0 and 80 hours per week (*M =* 18.03, *SD* = 14.07). Participants who consumed alcohol indicated they drank between 3 and 32 drinks per week (*M* = 10.27, *SD =* 6.62); participants who used recreational drugs indicated they use between 1 and 20 times per week (*M =* 3.40, *SD* = 4.81); and participants who smoked cigarettes indicated they consumed between 0 and 100 cigarettes per week (*M =* 30.60, *SD* = 29.32).

**TABLE 1:** Demographic variables of the sample of university students

Variable	Response	Counts	Percentage
Gender	Female	385	75.30
	Male	122	23.90
	Other	2	.39
Marital Status	Single	314	61.40
	Partnered	99	19.40
	Married	95	18.60
Born in Australia	Yes	416	81.40
	No	93	18.20
Language other than English at home	Yes	74	14.50
	No	434	84.90
Education (Highest attained)	High School (year 10)	33	6.50
	High School (year 12)	270	52.80
	Diploma (TAFE)	110	21.50
	Bachelor's degree	70	13.70
	Honours Degree	11	2.20
	Master's degree	12	2.30
	PhD	1	.20
Year of Study	1st Year	406	79.50
	2nd Year	55	10.80
	3rd Year	23	4.50
	4th Year	21	4.10
Study Mode	Internal	213	41.70
	External	58	11.40
	Internal and External	233	45.60
Study Load	Part‐time	142	27.80
	Fulltime	363	71.00
Employment Status	Unemployed	120	23.50
	Casual	179	35.00
	Part‐time	125	24.50
	Fulltime	83	16.20
Consume Alcohol	Yes	333	65.20
	No	176	34.40
Consume Recreational Drugs	Yes	81	15.90
	No	427	83.60
Smoke	Yes	66	12.90
	No	443	86.70

*Note:* *N* = 511; where percentage does not equal 100, demographic data was missing.

### Materials

#### The university needs instrument

The UNI comprises 30 items within six identified areas of need, each consisting of five items (see Appendix S1 for a full description of scale development). The six areas are *Academic needs,* which includes items related to academic staff and the development of academic skills (e.g., Lizzio et al., 2010; Walsh et al., 2010; Wibrowski et al., 2017); *Financial needs,* which includes items related to help affording study equipment, rent and groceries (e.g., Andrews & Wilding, 2004; Sharp & Theiler, 2018; Stewart et al., 1997; Walsh et al., 2010); *Family needs,* which includes items related to support from and spending time with family members (e.g., Beiter et al., 2015; Çivitci, 2015; Deb et al., 2015; Walsh et al., 2010; Wilcox et al., 2005); *Friend needs,* which includes items related to spending time with and social support from friends (e.g., Beiter et al., 2015; Çivitci, 2015; Deb et al., 2015; Robbins et al., 2004; Wilcox et al., 2005); *Practical needs,* which includes items related to sleep, transport to university and time management (e.g., Beiter et al., 2015; Chu‐Lien Chao, 2012; Çivitci, 2015; Deatherage et al., 2014; Lizzio et al., 2010; Lund et al., 2010; Robotham & Julian, 2006; Stewart et al., 1997; Walsh et al., 2010); *and Emotional needs,* which includes items related to stress, anxiety, depression, loneliness and frustration (Andrews & Wilding, 2004; Austin et al., 2010; Beiter et al., 2015; Chu‐Lien Chao, 2012; Çivitci, 2015; Deatherage et al., 2014; McKenzie & Schweitzer, 2001; Robotham & Julian, 2006; Sharp & Theiler, 2018; Stallman & Shochet, 2009; Stewart et al., 1997; Walsh et al., 2010; Wibrowski et al., 2017; Wilcox et al., 2005; Yan et al., 2014).

The sentence stem and response scale for the UNI were developed to be consistent with the wording established by previous psychosocial needs measures (e.g., Boyes et al., 2006; Choi et al., 2013; Patterson et al., 2014; Patterson et al., 2013; Patterson et al., 2019). That is, each of the 30 questions begins with the sentence stem ‘*I currently need*…’ (e.g., I currently need assistance to write academically). Participants then indicate, using a five‐point rating scale, how strongly they disagree or agree with each statement. The response scale was anchored by Strongly Disagree (1) and Strongly agree (5).

#### The Kessler psychological distress scale

The Kessler Psychological Distress Scale (K10; Kessler et al., 2002) is a valid and reliable measure (Andrews & Slade, 2008) of how often participants felt depressed and anxious within the previous 4 weeks. The K10 includes 10‐items and participants respond by indicating if they have experienced the symptom none of the time, a little of the time, some of the time, most of the time or all the time. The K10 score is based on the summed total of all items (Andrews & Slade, 2008). Within this study, we categorized distress levels based on the following recommendations low (10–15), moderate (16–21), high (22–29) and very high (30–50) psychological distress (Australian Bureau of Statistics, 2012). In our study, the K10 showed excellent generalizable reliability (Cronbach's α = .93; McDonald's ω = .93).

#### Demographic variables

To gain insight and generalizability of our sample, the following demographic variables were collected: Gender, marital status, if the participant was born in Australia, if the participant spoke a language other than English, the highest level of education, university attendance, current degree (undergraduate), study mode, year of study, study load, Grade Point Average (GPA) and employment status.

### Procedure

An Australian university human research ethics committee approved the cross‐sectional design to capture the psychosocial needs of undergraduate university students. Participants were recruited through the University of the Sunshine Coast's first‐year participation pool, Facebook posts, word of mouth and Cloud Research (*n* = 99). However, while most participants were recruited through the first‐year participation pool, the study was open to students enrolled in an undergraduate university degree at any year level (i.e., first, second, third or fourth year of study). Data was collected during the 2021 teaching calendar in Semester 1 and Semester 2.^1^ The first participants completed the survey during the third week of the semester (i.e., 13th March 2021), and the final participant completed the survey during week 2 of the second semester (i.e., 8th August 2021). During the first semester, 306 students completed the survey, and 127 participants completed the survey during the first week of session 2. A breakdown of the academic week participants completed the survey is provided in the Appendix S1.

Participants were provided with an information sheet attached to an announcement and a link to the Qualtrics survey. The first page of the Qualtrics survey also included the information statement, to ensure participants had read the instructions and not just followed the link. Participants were instructed that they could complete the survey using their computer or phone and that the survey should take 10–15 min to complete. After reading through the information sheet, participants consented to participate by clicking the ‘next button’. They were informed that any data they provided after that point might be used for data analysis. Participants then completed a series of questionnaires, which were randomized and counterbalanced across participants. All participants completed the demographic questions last. At the end of the survey, participants were provided with information about mental health support services for those who might benefit from additional support.

### Statistical analysis

#### Confirmatory factor analysis

Confirmatory Factor Analysis was used to test the factor structure of the UNI. As we aimed to confirm the theoretically proposed multifactor model of the UNI, CFA analysis was deemed more appropriate than a 1PL or 2PL IRT model, which are typically unidimensional and tend to focus on the scale and individual item characteristics. However, CFA conducted within a structural equation model framework is more concerned with the structural relations among observed and latent variables, such as confirming the factor structure of the UNI. As such, the R package Lavaan (Rosseel, 2012) was used to conduct Confirmatory Factor Analysis (CFA) using a diagonally‐weighted least‐squares estimation (Li, 2016). Global fit indices and the associated cut‐offs were used to estimate model fit (McDonald, 2010).

In our analysis, model fit was assessed using the comparative fit index (CFI), root mean square error of approximation (RMSEA), the Tucker Lewis Index (TLI), and the Standardized Root Mean Square Residual (SRMR). For the CFI, a value ≥.95 is recognized as an acceptable level of good fit (Hu & Bentler, 1999; Hooper et al., 2008; Kline, 2015). For RMSEA, lower non‐significant values are considered a better fit. In terms of cut‐off values, RMSEA values, ≤.07 are considered to indicate a good fit (Hooper et al., 2008; Steiger, 2007; Kline, 2015). Recommendations for the TLI indicate that values ≥.95 should be used to indicate a good model fit (Hooper et al., 2008; Kline, 2015). Finally, SRMR includes values ranging from 0 to 1, with lower values indicating better model fit (Byrne, 1998; Diamantopoulos & Siguaw, 2000; Hooper et al., 2008; Kline, 2015). While a value of zero indicates a perfect fit, an acceptable model fit is indicated by SRMR ≤.08 (Hu & Bentler, 1999; Kline, 2015). Non‐significant χ^2^ *p*‐values were another measure of model fit (Satorra & Bentler, 1994; Kline, 2015). However, it should be noted that when using robust maximum likelihood and diagonally weighted least‐squared models, χ^2^ test statistics tend to over‐reject models (i.e., significant χ^2^ *p*‐values; Li, 2016;).

In the first step, the hypothesized model was tested to confirm factor structure. For the CFA, a cut‐point for factor loadings was set at ± .50 (Field, 2013). Items that fell below this threshold were considered for removal. Items were also considered for removal if they did not significantly load on the expected theoretical factor. In the second step, models were tested to improve the model fit by adjusting parameters by observing modification indices. The parameters to be changed were determined by observing the expected change in model fit values (Saris et al., 2009; Kline, 2015). The strength of Pearson correlation coefficients was determined based on a meta‐analysis of psychological research (Gignac & Szodari, 2016). Gignac and Szodari (2016) conducted a meta‐analysis to empirically evaluate Cohen's correlation guidelines (i.e., small, *r =* .10; medium, *r =* .30; and large, *r =* .50). The meta‐analysis used a pool of 708 correlations testing individual difference research. Their results indicated that, in practice, *r* = .10, *r* = .20, *r* = .30 correspond with small, typical, and large effects in published psychological studies. Based on these empirical findings, effect sizes for correlations will be interpreted as small (*r* < ± .10), typical (*r* = ± .11 to ± .30) and large (*r* > ± .30).

## RESULTS

Missing data accounted for 8.1% of the total data and the results of Little's (1988) test suggested that data were missing completely at random, χ^2^ (509) = 450.98, *p* = .969. Missing data were imputed using the Expectation–Maximization (EM) algorithm (Kose, 2014; Rubin & Thayer, 1982). The EM imputation method was used because it tends to generate a more acceptable goodness of fit (Köse, 2014).

We have provided the descriptive statistics for each item in the Appendix S1. However, the top 10 psychosocial needs (percentage of Strongly Agree) from the UNI were the need to get more sleep, the opportunity to spend more time with my friends, to connect with other university students in my course, help to cope with feeling stressed, help to establish new friendships at university, help to cope with feeling anxious, assistance to afford text books for my classes, to feel that my family supports my study choice, the opportunity to spend more time with my family, help to cope with feeling lonely. However, it is important to note that all items were selected as strongly agree by at least 6.65% of the participants.

### Confirmatory factor analysis

#### Model fit

The hypothesized theoretical model was tested for model fit. Multivariate assumptions of normality were met. Skewness and Kurtosis statistics for all items were acceptable (i.e., < 2.00; Kim, 2013) and Mahalanobis distance for multivariate normality was acceptable, χ2 (30) = 29.94, *p* = .469. The initial analysis supported the hypothesized factor structure, demonstrating excellent model fit and met the criteria thresholds for RMSEA = .04, *p* = 1.00, CFI = .99, TLI = .99 and SRMR = .06. To test if the model might have been affected by including students from different year levels, the same analysis was conducted using only participants enrolled in their first year (*n* = 412). The model fit was also excellent and met the criteria thresholds for RMSEA = .04, *p* = 1.00, CFI = .99, TLI = .99 and SRMR = .06. However, as there is more explanatory power using the full sample, the following statistics utilize the data from all participants. Nonetheless, this suggests that the UNI is appropriate and may have utility for addressing the psychosocial needs of first‐year students.

#### Factor loadings

To confirm the factor structure of the UNI, parameter estimates were obtained for each item within each factor. The results factor loadings (see Table 2) show that each item is significantly loading on the relevant factor and in the correct direction. However, item 24 (i.e., assistance in enrolling in study) fell below the .50 threshold for factor loadings (Field, 2013). However, as the item showed convergent validity with the K10, was significantly loading on the factor and explained 17.7% of the variance in students' practical needs, the item was retained. Thus, no items were removed. Modification indices were also observed, and no items were identified to improve the model fit by contributing to an alternative factor. After examining the results of the CFA and measures of validity, all items within the UNI were retained. Therefore, the CFA has identified six multifaceted factors to measure psychosocial needs associated with academic support, financial support, support from family, support from friends, practical support and emotional support.

**TABLE 2:** Factor loading for each item within the theorized UNI factors

				95% CI			
Latent factor	Item	Standardized estimate	SE	Lower	Upper	Z	r^2^
Academic	1	.91	.03	.86	.96	36.96	.82
	2	.80	.03	.74	.86	26.98	.64
	3	.88	.03	.82	.94	27.13	.77
	4	.79	.03	.73	.85	25.47	.62
	5	.78	.03	.73	.84	26.11	.61
Financial	6	.69	.01	.66	.72	48.24	.48
	7	.85	.03	.80	.91	3.19	.72
	8	.85	.03	.80	.91	3.93	.73
	9	.86	.03	.81	.91	34.35	.75
	10	.86	.03	.81	.91	32.03	.74
Family	11	.73	.02	.70	.76	49.24	.53
	12	.72	.03	.67	.77	27.37	.52
	13	.58	.02	.53	.62	26.03	.33
	14	.72	.03	.67	.77	27.22	.51
	15	.52	.02	.48	.57	22.32	.27
Friends	16	.86	.02	.81	.90	38.52	.73
	17	.80	.03	.75	.85	3.07	.64
	18	.76	.02	.71	.81	31.49	.58
	19	.78	.03	.73	.84	29.84	.62
	20	.87	.03	.82	.93	31.59	.76
Practical	21	.77	.02	.74	.80	47.70	.60
	22	.74	.03	.68	.79	27.19	.54
	23	.63	.03	.57	.68	22.66	.39
	24	.43	.02	.39	.47	2.59	.19
	25	.64	.03	.58	.70	19.83	.41
Emotional	26	.81	.02	.77	.85	42.47	.65
	27	.81	.03	.76	.86	31.92	.65
	28	.78	.02	.74	.83	33.00	.61
	29	.83	.03	.78	.89	31.55	.70
	30	.82	.02	.77	.87	33.60	.67

*Note:* ^***^ *p* < .001.

#### Factor reliability and validity

The results in Table 3 indicate that there are significant covariance between all factors within the UNI. The covariance indicates that all factors move positively in the same direction. As expected, all factors are positively measuring a similar construct indicating psychosocial needs on one factor are associated with psychosocial needs on another factor. Each factor showed good to excellent internal reliability for academic needs, financial needs, family needs, friend's needs, practical needs and emotional needs. The reliability for the total scale was excellent (Cronbach's α = .94; McDonald's ω = .96; Hayes & Coutts, 2020; Viladrich et al., 2017).

**TABLE 3:** Factor covariance, reliability statistics (Cronbach's alpha and McDonald's omega) and correlations demonstrate convergent validity with the K10 and students' GPA

						Reliability		Correlations	
Factor	1	2	3	4	5	Alpha	Omega	K10	GPA
1. Academic						.90	.92	.30**	−.19**
2. Financial	.45					.92	.91	.30**	−.08
3. Family	.58	.47				.78	.79	.34**	−.14**
4. Friends	.53	.41	.59			.88	.91	.43**	−.18**
5. Practical	.59	.51	.61	.54		.76	.78	.47**	−.18**
6. Emotional	.48	.45	.6	.6	.64	.90	.90	.70**	−.29**

*Note:* *p < .05; **p < .01; **p < .001; K10 = Kessler Psychological distress scale; GPA = Grade Point Average.

#### Convergent validity with psychological distress

There was a high level of psychological distress within the sample (*M* = 26.39; *SD* = 8.96; 95% CI [25.58, 27.19]). Of the 511 participants, 35.6% (*n* = 182) reported very high distress, 31.1% (*n* = 159) reported high distress, 21.5% (*n* = 110) reported moderate distress, and 11.5% (*n* = 59) reported low distress. The results demonstrated that each factor within the UNI converged with students' levels of psychological distress (i.e., K10). That is, there was a significant positive relationship between students' levels of psychological distress and their academic needs, financial needs, family needs, friend needs, practical needs and emotional needs. These results provide convergent validity between psychological distress and psychosocial needs.

#### Academic performance and psychosocial needs

Within the sample, student's GPA was on average a credit (*M* = 3.33, *SD* = .99). However, there was a range of GPAs reported, including fail (*n* = 8; 1.6%), pass (*n* = 100; 19.6%), credit (*n* = 152; 29.70%), distinction (*n* = 164; 32.1%), and high distinction (*n* = 56; 11.0%), 31 (6.1%) participants did not provide their GPA.^2^Students' GPA was negatively associated with their psychosocial needs, except for their financial needs. These results show that students' academic needs, financial needs, family needs, friend needs and practical needs could be contributing to a reduction in their academic performance.

### Additional items

Of the 511 participants who completed the UNI, 64 provided suggestions for additional needs with a total of 111 suggestions. After analysing the additional needs, five independent reviewers determined that most of the suggestions were already covered in the UNI or were not relevant to the scope of the scale. However, some common themes were the need for consistency across courses (i.e., face‐to‐face and online), the need for motivation, to improve a sense of belonging, and diet.

## DISCUSSION

The current study aimed to develop and identify the factor structure of the university needs instrument and provide insights into the psychosocial needs of undergraduate university students. As expected, the CFA suggests that the items are measuring a similar underlying factor (i.e., psychosocial needs). The results supported the hypothesized six factors to measure the psychosocial needs of undergraduate university students: these relate to academic support, financial support, support from family, support from friends, practical support and emotional support.

### Psychometric properties of the UNI

The CFA shows that the UNI has sound psychometric properties and the items within each factor can significantly measure the theorized latent factors (i.e., academic support, financial support, support from family, support from friends, practical support and emotional support). Further, our analysis indicates that all latent factors covary with one another and in the same positive direction. The direction and strength of the covariance provide evidence that all factors are measuring a similar higher‐order factor (i.e., psychosocial needs). The covariance also show that the items are contributing to measuring a multifaceted construct associated with the different types of psychosocial needs experienced by undergraduate university students. While our study did not test higher‐order factors, future research should aim to identify if the UNI is significantly measuring a higher‐order latent factor. We theorize that the higher‐order factor is related to psychosocial needs more broadly.

### University student needs

We have confirmed the findings of Tinto's model of academic persistence (1993, 2012) by identifying that there are specific academic and social needs of students. These findings also show that students have very specific academic and social support needs that have not been previously considered within the retention literature. Further research will need to validate if students with fewer psychosocial needs (i.e., positive interaction and integration at university) are more likely to remain at university. Nonetheless, our study has identified a unique subset of academic and social needs and a more diverse range of psychosocial needs that are potentially contributing to lower GPAs and higher levels of psychological distress. The information within the UNI could be used by tertiary institutions to provide targeted psychological and social support for students to create a positive academic and social integration within student cohorts.

The UNI has identified a range of psychosocial needs experienced by university students. Undergraduate university students indicated they currently needed emotional support. Our results confirm that undergraduate students commonly report feelings of anxiousness, loneliness, frustration and depression (Andrews & Wilding, 2004; Beiter et al., 2015; Gollust et al., 2007; Sharp & Theiler, 2018) with all emotional items loading strongly together. This finding is unsurprising, given research within vulnerable populations shows that emotional support is related to a reduction in psychological distress (Boudreault‐Bouchard et al., 2013; Griffith, 1984; Krycak et al., 2012; McLuckie et al., 2018; Sharp & Theiler, 2018). Nonetheless, it remains clear that university students are not only experiencing high to very high levels of psychological distress, but they need help with dealing with their feelings of stress, anxiousness, frustration, depression and loneliness.

Financial support was a strong factor for university students, with most students indicating they needed financial assistance. About one in three participants agreed that they currently need assistance to afford textbooks. These results may support findings indicating a link between the financial burdens of attending university and elevated levels of stress (Walsh et al., 2010). Together, this indicates that the financial burden of students attending university is a significant issue and could have implications for their psychological well‐being. Further consideration should be given to addressing the burden of costs associated with purchasing textbooks and essential equipment needed to study at university.

The factor structure of the UNI showed that support from friends and family seems to offer different types of social support. This finding suggests that providing, having access to, and being able to communicate with family members or peers is an important psychosocial need for groups experiencing high levels of psychological distress. The separation of family and friend support indicates that the type of social support offered by these two groups differs. For example, the buffering hypothesis explains that the social support received from family members might intervene between the stressful situation and the individual (Cohen & Wills, 1985; Wilcox et al., 2005). Secondly, the direct effects hypothesis suggests that support from friends and peers might enable an individual to have access to support from others going through a similar experience, but also provides individuals with a greater sense of control by normalizing their circumstances (Cohen & Wills, 1985; Wilcox et al., 2005). We argue that family support can act as a buffer between psychosocial stressors and the student, while the support provided by friends normalizes their circumstances.

The practical needs of university students were also confirmed as a psychosocial factor. Indeed, a high proportion of students strongly agreed that they need more sleep supporting the extensive literature identifying the poor‐quality sleep patterns of undergraduate students (e.g., Campbell et al., 2018; Vedaa et al., 2019). This finding suggests that students' subjective perception of their need for more sleep is positively associated with their levels of psychological distress. However, the practical needs factor does seem to be the weakest, in terms of reliability and strength of the factor loadings. Further consideration should be given to identifying the robustness of the practical needs factor and the relevancy of the items to measure psychosocial needs. For instance, this is reflected in the correlations between psychosocial needs and academic week (i.e., the week of the session).^3^ Indeed, practical needs were the only factor that correlated significantly with the academic week and suggests that as the semester progresses students do not need as much assistance with managing their study load, timetables, enrolments and transport. However, this finding is somewhat consistent with Reeves and Maloney (2017) who showed that most students develop the coping skills to manage the study demands of the university by the second semester. These findings suggest that students do have several practical needs, however, these needs might be exacerbated at the beginning of the semester, rather than at the end of the semester.

Consistent with the psychosocial needs literature (e.g., Fiori & Consedine, 2013; McDonald et al., 2016), our results suggest that providing university students with support services aimed at helping them deal with their feelings of loneliness, anxiety, depression, stress and frustration, could help reduce psychological distress. Indeed, emotional support has been effective at reducing psychological distress among vulnerable at‐risk populations; including adolescents, cancer patients and minority groups (e.g., Hovey & Magaña, 2002; Jacobsen & Jim, 2008; McDonald et al., 2016).

### Limitations

The study was not without limitations, and these must be considered when evaluating the validity of the scale. First, most of the data collected for this study included participants from a single country who were largely first‐year psychology students. As such, the results might not be representative and generalizable to all university students. We recommend that future studies recruit participants from multiple countries and cultures to provide a more heterogeneous sample and improve the generalizability of the findings for the use of UNI to measure the psychosocial needs of undergraduate university students.

Second, because the study was open to students enrolled in their first, second, third, and fourth years of study, the UNI might not be capturing the unique needs associated with each year level. As such, the utility of the scale should be further confirmed by validating the scale within different year groups. Indeed, we encourage future validations of the UNI to conduct invariance analysis among the different years—we acknowledge that this will require a substantial sample size but would provide further empirical support for the validity of and importance of psychosocial needs for all undergraduate students.

A third limitation could be the wording of the sentence stem for the scale we have used. For example, because we ask students what they currently need, ‘current needs’ might differ depending on the point in the academic year that the questionnaire is administered. However, as the scale's utility is intended to provide targeted psychosocial support to university students, it is necessary to identify what students currently need. Also, the sentence STEM ‘*I currently need*’ is consistent with existing psychosocial needs measures (Chan et al., 2020; McDonald et al., 2020; Patterson et al., 2013, Patterson et al., 2014, Patterson et al., 2017; Patterson et al., 2020; Varathakeyan et al., 2018; Williams, 2012). Nonetheless, our findings provide initial evidence for the use of the UNI to identify the psychosocial needs of undergraduate university students.

### Future directions

The CFA shows that UNI has sound psychometric properties and the items within each factor can significantly measure the theorized latent factors (i.e., academic support, financial support, support from family, support from friends, practical support and emotional support). Further, our analysis indicates that all latent factors covary with one another and in the same positive direction. The direction and strength of the covariance provide some evidence that all factors might be measuring a similar higher‐order factor (i.e., psychosocial needs). The covariance also show that the items are contributing to measuring a multifaceted construct associated with the different types of psychosocial needs experienced by undergraduate university students. While our study did not test higher‐order factors, future research should aim to identify if the UNI is significantly measuring a higher‐order latent factor. We theorize that the higher‐order factor is related to psychosocial needs more broadly.

## CONCLUSION

Our findings provide initial evidence that undergraduate university students have a range of psychosocial needs associated with dealing with emotions, assistance with finances, support from friends and family, assistance developing academic skills and support from academic staff. While we have only provided an initial validation, we recommend that universities use the UNI to help screen and identify the psychosocial needs of their students and in turn identify appropriate support which could help reduce their psychological distress, improve their psychosocial well‐being and increase academic performance.

## ACKNOWLEDGEMENT

Open Access Funding provided by University of the Sunshine Coast within the CAUL Agreement.

## CONFLICT OF INTEREST

The authors declare that they have no conflict of interest.

## AUTHOR CONTRIBUTIONS

**Richard Tindle:** Conceptualization; Data curation; Formal analysis; Methodology; Project administration; Resources; Writing – original draft; Writing – review & editing. **Paola Castillo:** Methodology; Project administration; Writing – original draft; Writing – review & editing. **Natalie Doring:** Writing – original draft; Writing – review & editing. **Leigh Grant:** Writing – original draft; Writing – review & editing. **Royce Willis:** Writing – original draft; Writing – review & editing.

## INFORMED CONSENT

Informed consent was obtained from all individual adult participants included in the study.

## COMPLIANCE WITH ETHICAL STANDARDS

All procedures performed in studies involving human participants were in accordance with the ethical standards of the institutional research committee and with the 1964 Helsinki Declaration and its later amendments or comparable ethical standards.

## Data Availability

The data that support the findings of this study are openly available through Mendeley data https://data.mendeley.com/drafts/4x8f48b6jb

## Footnotes

^1^
The data was collected during the Covid‐19 pandemic, which could have impacted on students needs, distress, and the subsequent development of the scale. Further validation of the scale should be conducted post‐pandemic.

^2^
Data were MCAR. Little's test was non‐significant χ^2^ < .001, *p* = 1.000.

^3^
Bivariate Pearson correlations showed no correlation between academic week and students psychosocial needs for Academic needs (r = −.06, *p* = .237), financial needs (r = −.05, *p* = .324), family needs (r = −.03, *p* = .288), nor emotional needs (r = −.05, *p* = .326). However, practical needs were significantly and negatively associated with the academic week (r = −.17, *p* < .001).
