# Supplementary material for: Developing and validating a university needs instrument to measure the psychosocial needs of university students
Source: Br J Educ Psychol. 2022 May 19;92(4):1550–70. doi: 10.1111/bjep.12515 (PMC9790289; doi:10.1111/bjep.12515)
Supplement: Supplementary file 1 [file BJEP-92-1550-s001.docx]

**Supplementary Materials**

### Development of the University Needs Instrument

The factors were first identified by reviewing developed and validated psychosocial needs instruments used within at-risk groups (e.g., Clinton-McHarg et al., 2012; Girgis et al., 2011; Patterson, Pearce, et al., 2011). The items that comprise the University Needs Instrument (UNI) were then developed by the researchers of this paper. Development of items was created independently by the researchers of this paper each item that was developed needed to be empirically supported for the item as a need and was required to have empirical support (from multiple studies) to confirm it was a psychosocial need and related to students’ distress, wellbeing, or academic performance.

After individual items were developed, they were independently reviewed by each member of the research team. Each item was then assigned to one of 6 factors (academic need, financial need, family need, friend need, practical need, or emotional need). The factors and their associated items were then independently reviewed by all members of the research team before the final questionnaire was developed. The attached supplementary material presents each item and the references to support the inclusion of the item in the scale. Please note, that this is not an exhaustive list of all possible references that can be used to support the items within the UNI. For example, there is a plethora of research to support that university students experience stress, anxiety, and depression and including all of these was not practical. Nonetheless, each item developed within the UNI was based on empirical evidence and has been identified as a psychosocial need associated with university students’ distress, wellbeing, or academic performance

We would also like to acknowledge that the development of the scale underwent initial revisions at the British Journal of Educational Psychology which led to further development of the scale. The response scale used for the UNI was amended based on three independent reviewers and an Editor at the British Journal of Educational Psychology. For example, the initial response scale asked participants to indicate if the item was a current need or a non-current need by using the scale ‘no need’, ‘low need’ ‘moderate need, ‘very high need", and "high need". However, based on the analysis conducted (e.g., an exploratory factor analysis) and revision suggestion, the response scale was deemed to be logically/grammatically incorrect and statistically unbalanced. Based on these suggestions, the appropriate response scale for the items was changed to a five-point Likert scale with responses ranging from Strongly Disagree to Strongly Agree.

***Table 1.***

*Literature used to inform the development of the University Needs Instrument*

| **No.** | **Factor** | **Item** | **Reference for item** |
| --- | --- | --- | --- |
| 1 | Academic | to feel supported by my Lecturers | Edwards, et al., 2016; Furnham & Chamorro-Premuzic, 2005; Lizzio et al., 2002; Neves, 2019; Tinto 1993; Tinto, 2012; Radloff & Coates, 2009; Richardson, 2011; Wilcox et al., 2006. |
| 2 | Academic | to feel supported by my Tutors | Furnham & Chamorro-Premuzic, 2005; Krumrei-Mancuso, et al., 2013; Lizzio et al., 2002; Neves, 2019; Radloff & Coates, 2009; Retna, et al., 2009; Richardson, 2011; Wilcox et al., 2006. |
| 3 | Academic | additional support to understand the course material | Dukhan 2020; Edwards, et al., 2016; Johnston Salaz 2019; Lizzio et al., 2002; Nordin 2018; Tinto 1993; Tinto, 2012; Stander 2020; Wilcox et al., 2006. |
| 4 | Academic | assistance in developing academic skills | Dukhan 2020; Johnston Salaz 2019; Lizzio et al., 2002; Nordin 2018; Stander 2020; Wibrowski et al., 2017. |
| 5 | Academic | assistance to write academically | Dukhan 2020; Ebadi Rahimi 2019; Elliott et al., 2019; Hussien 2020; Johnston Salaz 2019; Lizzio et al., 2002; Mitchell et al., 2021; Nordin 2018; Stander 2020; Wibrowski et al., 2017. |
| 6 | Financial | assistance to pay rent | Alamel, 2021; Andrews, & Wilding, 2004; Fyall et al., 2019; Khosla et al., 2020; Martinez et al., 2021; Sharp & Theiler, 2018; Stewart et al., 1997; Walsh et al, 2010; Ruming & Dowling, 2017. |
| 7 | Financial | assistance to buy groceries | Andrews, & Wilding, 2004; Khosla et al., 2020; Sharp & Theiler, 2018; Stewart et al., 1997; Walsh et al, 2010. |
| 8 | Financial | assistance to pay for utilities (water, electricity, internet) | Andrews, & Wilding, 2004; Khosla et al., 2020; Sharp & Theiler, 2018; Stewart et al., 1997; Walsh et al, 2010. |
| 9 | Financial | assistance to afford textbooks for my classes | Andrews, & Wilding, 2004; Clinton et al., 2019; Grilmandi, 2019; Khosla et al., 2020; Sharp & Theiler, 2018; Smith et al., 2016; Stewart et al., 1997; Okamoto, 2013; Mills, 2019; Universities Australia, 2018; USPIRG, 2014; Walsh et al, 2010. |
| 10 | Financial | assistance to afford study equipment (e.g., laptop, pens, paper) | Andrews, & Wilding, 2004; Khosla et al., 2020; Sharp & Theiler, 2018; Stewart et al., 1997; Walsh et al, 2010. |
| 11 | Family | help dealing with family pressure to succeed | Alaraj 2019; Çivitci, 2015; Chiwuzie, 2021; Deb et al, 2015; Khan & Khadija, 2019; Metzger et al., 2017; Oluwole & Oyedun, 2014; Reis et al., 2021; Yehuda & Khaldy 2013. |
| 12 | Family | help adjusting my lifestyle to suit attending university | Beiter et al, 2015; Böke et al., 2019; Denovan & Macaskill, 2013; Gale & Thalitaya, 2015; Hoffman, 2021; Julia, 2012; Leung 2017; Malau-Aduli et al, 2021; Mudhovozi, 2017; Suprapto 2019; Truschel and Jan Yau et al., 2012; Wada et al., 2016; Walsh et al, 2010. |
| 13 | Family | to feel that my family supports my study choice | Beiter et al, 2015; Truschel & Hoffman, 2021; Walsh et al, 2010. |
| 14 | Family | emotional support from family members | Beiter et al, 2015; Truschel & Hoffman, 2021; Walsh et al, 2010; Wilcox et al., 2006. |
| 15 | Family | the opportunity to spend more time with family | Alaraj 2019; Beiter et al, 2015; Çivitci, 2015; Chiwuzie, 2021; Deb et al, 2015; Khan & Khadija, 2019; Reis et al., 2021; Metzger et al., 2017; Oluwole & Oyedun, 2014; Yehuda & Khaldy 2013. |
| 16 | Friends | the opportunity to spend more time with friends | Alsubaie et al., 2019; Al-Gamal & Long, 2012; Beiter et al, 2015; Çivitci, 2015; Deb et al, 2015; Gfellner & Ana, 2011; Granieri, 2021; Hamdan-Mansour et al., 2014; Lai & Ma, 2016; Lefevor et al., 2018; Løvseth et al., 2020; Stallman, 2008; Ratelle et al., 2013; Robotham & Julian, 2006; Segrin et al., 2016; Steele, et al., 2005; van Rhijn et al., 2016; Talwar & Fadzil, 2013; Vungkhanching, et al., 2017; Wilcox et al., 2005. |
| 17 | Friends | to connect with other university students in my course | Arslan et al., 2014; Awanf et al., 2014; Hagler et al., 2021; Johnson et al., 2018; McAllister et al., 2014; Kaakinen et al., 2017; Kamardeen & Sunindijo, 2018; Karagiannopoulou & Milienos, 2018; Kuittinen & Merila¨inen, 2011; Robbins et al., 2004; Travaglino, 2020; Räisänen, et al., 2016; Zhu et al, 2013. |
| 18 | Friends | help to establish new friendships at university | Akacan 2017; Çivitci, 2015; Gökdag, 2015; Robbins et al., 2004; Salami, 2011; Sevinç & Gizir, 2014; Thurber & Walton, 2012; Tsitsas et al., 2019; Tamannaeifar & Shahmirzaei, 2019; Wilcox et al., 2006. |
| 19 | Friends | emotional support from my friends not at univeristy | Çivitci, 2015; Dhurup, 2016; Granieri et al., 2021; Hautasaari et al., 2017; Liftiana & Fakhruddiana, 2019; Robbins et al., 2004; Satoshi, 2021; Strnadová et al., 2020; Sun et al, 2018; Wilcox et al., 2006; Xerri, et al., 2018; Zyl and Ahmed et al., 2011. |
| 20 | Friends | support from friends in the same course as me | Arslan et al., 2014; Awanf et al., 2014; Hagler et al., 2021; Johnson et al., 2018; Kaakinen et al., 2017; Kamardeen & Sunindijo, 2018; Karagiannopoulou & Milienos, 2018; Kuittinen & Merila¨inen, 2011; McAllister et al., 2014; Räisänen, et al., 2016; Robbins et al., 2004; Travaglino, 2020; Zhu et al, 2013. |
| 21 | Practical | assistance in managing my study load | Beiter et al, 2015; Chu-Lien Chao, 2012; Çivitci, 2015; Deatherage, Lizzio et al., 2002; Lund et al, 2010; Omar et al., 2020; Reeves & Maloney, 2017; Robotham & Julian, 2006; Servaty-Seib, & Aksoz, 2014; Stewart et al., 1997; Walsh et al, 2010. |
| 22 | Practical | assistance in managing my timetable | Beiter et al, 2015; Chu-Lien Chao, 2012; Çivitci, 2015; Deatherage, Servaty-Seib, & Aksoz, 2014; Lizzio et al., 2002; Lund et al, 2010; Omar et al., 2020; Reeves & Maloney, 2017; Robotham & Julian, 2006; Salih, et al, 2021; Stewart et al., 1997; Walsh et al, 2010. |
| 23 | Practical | assistance in enrolling in study units | Beiter et al, 2015; Chu-Lien Chao, 2012; Çivitci, 2015; Deatherage, Servaty-Seib, & Aksoz, 2014; Lizzio et al., 2002; Lund et al, 2010; Omar et al., 2020; Robotham & Julian, 2006; Salih et al., 2021; Stewart et al., 1997; Walsh et al, 2010. |
| 24 | Practical | to get more sleep | Abdulghani, 2012; Almojali, 2017; Asaad et al., 2014; Beiter et al., 2015; Brick et al., 2010; Campbell et al., 2018; Chu-Lien Chao, 2012; Çivitci, 2015; Deatherage, Servaty-Seib, & Aksoz, 2014; Digdon & Landry; 2013; Doolin et al., 2018; Eskin et al., 2016; Gaultne, 2010; Gomes et al., 2011; Gupta et al., 2016; Gilbert & Weavewr et al., 2010; John-Henderson et al., 2017; Kabrita & Hajjar-Muça, 2016; Kandari et al., 2017; Khader et al., 2020; Lawson et al., 2019; Lemma et al., 2012; Lemma et al., 2014; Lo´pez-Rodrı´guez et al., 2017; Lund et al, 2010; McGillivray & Pidgeon, 2015; Mirghani et al., 2015; Orzech et al,. 2017; Peltzer & Pengpid, 2015; Robotham & Julian, 2006; Rodgers et al., 2016; Schlarb et al., 2017; Seun-Fadipe et al., 2017; Vedaa et al., 2019; Zhou et al., 2014. |
| 25 | Practical | assistance with transport to university | Crespo et al., 2012; Metzger et al., 2017; Salih, et al, 2021; Silinda & Brubacher, 2016; Walsh et al, 2010. |
| 26 | Emotional | help with feeling stressed | Andrews & Wilding, 2004; Asif et al., 2020; Austin, Saklofske, & Mastoras, 2010; Bedewy & Gabriel., 2015; Beiter et al., 2015; Besser & Zeigler-Hill, 2014; Böke et al., 2019; Cavallo et al., 2016; Cheung et al., 2020; Chu-Lien Chao, 2012; Çivitci, 2015; Deatherage, Servaty-Seib, & Aksoz, 2014; Debowska et al., 2020; Feeney, Hussey, & Donnellan, 2010; Gallego et al., 2014; Gritsenko et al., 2020; Husk et al., 2020; Lovell et al., 2015; Othman et al., 2019; Regehr et al., 2013; Ribeiro et al., 2018; Rith-Najaria et al., 2019; Robotham & Julian, 2006; Stewart et al., 1997; Shamsuddin et al., 2013; Walsh & Clinciu, 2013. |
| 27 | Emotional | help with feeling anxious | Asif et al., 2020; Cheung et al., 2020; Chu-Lien Chao, 2012; Çivitci, 2015; Deatherage et al., 2014; Debowska et al., 2020; Farrer et al., 2016; Gallego et al., 2014; Gritsenko et al., 2020; Husk et al., 2020; Islam et al., 2020; Lovell et al., 2015; Pidgeon et al., 2014; Rith-Najaria et al., 2019; Robotham & Julian, 2006; Sharp & Theiler, 2018; Stallman & Shochet, 2009; Stewart et al., 1997; Wibrowski, Matthews, & Kitsantas, 2017; Wilcox et al., 2005. |
| 28 | Emotional | help with feeling lonely | Alkan, 2014; Aradhana & Rupali, 2017; Beiter et al, 2015; Bernardon et al., 2011; Chang, 2012; Çivitci, 2015; Deatherage et al., 2014; Ozsaker et al., 2015; Rahman et al., 2012; Thomas et al., 2020; Tian et al., 2018; Walsh et al., 2010; Wilcox, Winn & Fyvie-Gauld, 2005; Yıldız et al., 2017. |
| 29 | Emotional | help with feeling frustrated | Al-Duba et al., 2010; Awang et al., 2014; Bautista et al., 2018; Chu-Lien Chao, 2012; Khanehkeshi & Basavarajappa, 2011; Madhyastha et al., 2015; Walsh et al, 2010. |
| 30 | Emotional | help with feeling depressed | Alsubaie etal, 2019; Al-Qaisy, 2011; Asif et al., 2020; Beiter et al, 2015; Chen et al., 2013; Cheung et al., 2020; Chu-Lien Chao, 2012; Çivitci, 2015; Deatherage et al., 2014; Debowska et al., 2020; Demirci et al., 2015; Farrer et al., 2016; Gallego et al., 2014; Ibrahim et al., 2013; Islam et al., 2020; Khawaja et al., 2013; Lei et al., 2016; Lovell et al., 2015; McKenzie & Schweitzer, 2001; Othieno et al., 2014; Pidgeon et al., 2014; Rith-Najaria et al., 2019; Robotham & Julian, 2006; Said et al., 2013; Shamsuddin et al., 2013; Yan et al., 2014. |

**Table 2.**

*Distribution of when participants completed the survey relative to the academic week within semester 1 and Semester 2.*

|  | Semester 1 | | Semester 2 | |
| --- | --- | --- | --- | --- |
| Semester/Week | N | % | N | % |
| Week 1 | ­- | - | 127 | 24.9 |
| Week 2 | 2 | 0.5 | - | - |
| Week 3 | 33 | 7.6 | 10 | 4.9 |
| Week 4 | 22 | 5.1 | 15 | 7.3 |
| Week 5 | 9 | 2.1 | 5 | 2.4 |
| Week 6 | 7 | 1.6 | 7 | 3.4 |
| Week 7 | 12 | 2.8 | 5 | 2.4 |
| Week 8 | 12 | 2.8 | 6 | 2.9 |
| Week 9 | 12 | 2.8 | 2 | 1.0 |
| Week 10 | 24 | 5.5 | 9 | 4.4 |
| Week 11 | 28 | 6.5 | 19 | 9.3 |
| Week 12 | 41 | 9.5 | - | - |
| Week 13 | 59 | 13.6 | - | - |
| Week 14 | 45 | 10.4 | - | - |

**Table 3**

*Descriptive statistics, confidence intervals, for each UNI item, and the percentage of times each response was selected for each item.*

|  |  |  |  | 95% CI | |  | Correlations | |  | Response Choice (SD - SA) | | | | |
| --- | --- | --- | --- | --- | --- | --- | --- | --- | --- | --- | --- | --- | --- | --- |
| Factor | Item | *M* | *SD* | Lower | Upper |  | Academic Week | K10 |  | 1 | 2 | 3 | 4 | 5 |
|  | *I currently need…* |  |  |  |  |  |  |  |  |  |  |  |  |  |
|  | **Academic** |  |  |  |  |  |  |  |  |  |  |  |  |  |
| Academic | …to feel supported by my Lecturers | 2.93 | 1.10 | 2.84 | 3.03 |  | -.05 | .202** |  | 13.50% | 25.24% | 39.14% | 14.29% | 7.83% |
| Academic | …to feel supported by my Tutors | 2.99 | 1.13 | 2.90 | 3.09 |  | -.06 | .222** |  | 10.18% | 19.18% | 43.84% | 18.79% | 8.02% |
| Academic | …additional support to understand the course material | 2.78 | 1.10 | 2.68 | 2.87 |  | -.06 | .320** |  | 38.55% | 28.96% | 17.22% | 8.61% | 6.65% |
| Academic | …assistance in developing academic skills | 2.95 | 1.05 | 2.86 | 3.04 |  | -.03 | .259** |  | 17.61% | 21.53% | 24.46% | 23.09% | 13.31% |
| Academic | …assistance to write academically | 2.89 | 1.09 | 2.79 | 2.98 |  | -.08 | .258** |  | 28.18% | 26.42% | 21.53% | 14.68% | 9.20% |
|  | **Financial** |  |  |  |  |  |  |  |  |  |  |  |  |  |
| Financial | …assistance to pay rent | 2.40 | 1.45 | 2.27 | 2.52 |  | -.05 | .217** |  | 32.88% | 20.74% | 18.20% | 16.83% | 11.35% |
| Financial | …assistance to buy groceries | 2.39 | 1.37 | 2.27 | 2.51 |  | -.08 | .305** |  | 29.94% | 17.81% | 17.61% | 17.81% | 16.83% |
| Financial | …assistance to pay for utilities | 2.40 | 1.40 | 2.28 | 2.52 |  | -.04 | .244** |  | 37.18% | 21.14% | 18.00% | 12.92% | 10.76% |
| Financial | …assistance to afford textbooks for my classes | 2.74 | 1.47 | 2.61 | 2.87 |  | -.06 | .289** |  | 37.96% | 20.35% | 16.83% | 13.31% | 11.55% |
| Financial | …assistance to afford study equipment | 2.53 | 1.39 | 2.41 | 2.65 |  | -.02 | .272** |  | 40.31% | 19.18% | 13.11% | 15.07% | 12.33% |
|  | **Family** |  |  |  |  |  |  |  |  |  |  |  |  |  |
| Family | …help dealing with family pressure to succeed | 2.64 | 1.37 | 2.52 | 2.75 |  | -.05 | .413** |  | 12.13% | 20.74% | 41.88% | 16.63% | 8.61% |
| Family | …help to adjust my lifestyle to suit attending university | 3.02 | 1.25 | 2.91 | 3.13 |  | .06 | .313** |  | 58.51% | 19.96% | 10.37% | 4.50% | 6.65% |
| Family | …to feel that my family supports my study choice | 2.92 | 1.40 | 2.80 | 3.04 |  | -.02 | .146** |  | 13.11% | 17.22% | 26.03% | 28.96% | 14.68% |
| Family | …emotional support from family members | 3.15 | 1.25 | 3.04 | 3.26 |  | -.03 | .290** |  | 13.31% | 19.57% | 27.79% | 26.03% | 13.31% |
| Family | …the opportunity to spend more time with my family | 3.25 | 1.21 | 3.15 | 3.36 |  | -.111* | .097* |  | 14.87% | 19.37% | 27.59% | 25.05% | 13.11% |
|  | **Friends** |  |  |  |  |  |  |  |  |  |  |  |  |  |
| Friends | …the opportunity to spend more time with my friends | 3.44 | 1.17 | 3.34 | 3.54 |  | .00 | .330** |  | 13.70% | 18.79% | 22.11% | 27.01% | 18.40% |
| Friends | …to connect with other university students in my course | 3.27 | 1.27 | 3.16 | 3.38 |  | -.02 | .335** |  | 25.24% | 25.05% | 17.81% | 17.42% | 14.48% |
| Friends | …help to establish new friendships at university | 3.13 | 1.34 | 3.02 | 3.25 |  | -.04 | .361** |  | 15.85% | 23.48% | 27.79% | 18.00% | 14.87% |
| Friends | …emotional support from my friends not at university | 3.06 | 1.23 | 2.96 | 3.17 |  | .00 | .368** |  | 23.68% | 21.92% | 20.55% | 18.79% | 15.07% |
| Friends | …support from friends in the same course as me | 3.06 | 1.25 | 2.95 | 3.17 |  | -.088* | .366** |  | 9.98% | 16.63% | 23.09% | 31.51% | 18.79% |
|  | **Practical** |  |  |  |  |  |  |  |  |  |  |  |  |  |
| Practical | …assistance in managing my study load | 2.93 | 1.30 | 2.82 | 3.04 |  | -.02 | .358** |  | 27.59% | 23.68% | 18.79% | 17.42% | 12.52% |
| Practical | …assistance in managing my timetable | 2.50 | 1.29 | 2.39 | 2.61 |  | -.05 | .392** |  | 15.66% | 18.59% | 21.33% | 25.64% | 18.79% |
| Practical | …assistance in enrolling in study units | 2.16 | 1.22 | 2.05 | 2.26 |  | -.167** | .299** |  | 13.11% | 21.33% | 26.22% | 25.05% | 14.29% |
| Practical | …to get more sleep | 3.49 | 1.30 | 3.38 | 3.61 |  | -.116** | .336** |  | 10.96% | 14.87% | 27.59% | 31.31% | 15.26% |
| Practical | …assistance with transport to university | 1.81 | 1.20 | 1.70 | 1.91 |  | -.210** | .278** |  | 7.44% | 13.50% | 27.20% | 31.70% | 20.16% |
|  | **Emotional** |  |  |  |  |  |  |  |  |  |  |  |  |  |
| Emotional | …help to cope with feeling stressed | 3.32 | 1.24 | 3.22 | 3.43 |  | -.02 | .555** |  | 11.74% | 15.66% | 25.83% | 27.40% | 19.37% |
| Emotional | …help to cope with feeling anxious | 3.18 | 1.31 | 3.06 | 3.29 |  | -.05 | .613** |  | 10.96% | 21.72% | 39.92% | 17.81% | 9.59% |
| Emotional | …help to cope with feeling lonely | 2.80 | 1.39 | 2.68 | 2.92 |  | -.05 | .550** |  | 11.15% | 19.57% | 39.33% | 18.59% | 11.35% |
| Emotional | …help to cope with feeling frustrated | 2.93 | 1.28 | 2.81 | 3.04 |  | -.08 | .585** |  | 23.48% | 15.85% | 21.53% | 23.68% | 15.46% |
| Emotional | …help to cope with feeling depressed | 2.71 | 1.39 | 2.59 | 2.83 |  | -.03 | .667** |  | 9.98% | 14.09% | 20.35% | 27.79% | 27.79% |
| **p<.05; **p<.01; *** p < .001; K10 = Kessler 10 psychological distress scale; SD = Strongly Disagree; SA = Strongly Agree* | | | | | | | | |  |  |  |  |  |  |

**The University Needs Instrument**

| ***Items*** | *1*  *Strongly Disagree* | *2* | *3* | *4* | *5*  *Strongly Agree* |
| --- | --- | --- | --- | --- | --- |
| **Academic support needs** |  |  |  |  |  |
| 1. …to feel supported by my Lecturers |  |  |  |  |  |
| 1. …to feel supported by my Tutors |  |  |  |  |  |
| 1. …additional support to understand the course material |  |  |  |  |  |
| 1. …assistance in developing academic skills |  |  |  |  |  |
| 1. …assistance to write academically |  |  |  |  |  |
| **Financial support needs** |  |  |  |  |  |
| 1. …assistance to pay rent |  |  |  |  |  |
| 1. …assistance to buy groceries |  |  |  |  |  |
| 1. …assistance to pay for utilities |  |  |  |  |  |
| 1. …assistance to afford textbooks for my classes |  |  |  |  |  |
| 1. …assistance to afford study equipment |  |  |  |  |  |
| **Family support needs** |  |  |  |  |  |
| 1. …help to deal with family pressure to succeed |  |  |  |  |  |
| 1. …help to adjust my lifestyle to suit attending university |  |  |  |  |  |
| 1. …to feel that my family supports my study choice |  |  |  |  |  |
| 1. …emotional support from family members |  |  |  |  |  |
| 1. …the opportunity to spend more time with my family |  |  |  |  |  |
| **Friend support needs** |  |  |  |  |  |
| 1. …the opportunity to spend more time with my friends |  |  |  |  |  |
| 1. …to connect with other university students in my course |  |  |  |  |  |
| 1. …help to establish new friendships at university |  |  |  |  |  |
| 1. …emotional support from my friends not at university |  |  |  |  |  |
| 1. …support from friends in the same course as me |  |  |  |  |  |
| **Practical support needs** |  |  |  |  |  |
| 1. …assistance in managing my study load |  |  |  |  |  |
| 1. …assistance in managing my timetable |  |  |  |  |  |
| 1. …assistance in enrolling in study units |  |  |  |  |  |
| 1. …to get more sleep |  |  |  |  |  |
| 1. …assistance with transport to university |  |  |  |  |  |
| **Emotional support needs** |  |  |  |  |  |
| 1. …help to cope with feeling stressed |  |  |  |  |  |
| 1. …help to cope with feeling anxious |  |  |  |  |  |
| 1. …help to cope with feeling lonely |  |  |  |  |  |
| 1. …help to cope with feeling frustrated |  |  |  |  |  |
| 1. …help to cope with feeling depressed |  |  |  |  |  |

**References**

Abdulghani, H. M., Alrowais, N. A., Bin-Saad, N. S., Al-Subaie, N. M., Haji, A. M. A., & Alhaqwi, A. I. (2012). Sleep disorder among medical students: Relationship to their academic performance. *Medical Teacher*, *34*(SUPPL. 1). https://doi.org/10.3109/0142159X.2012.656749

Alamel, A. (2021). The magnitude of “all-inclusive energy packages” in the UK student housing sector. *Area*, *53*(3), 464–472. https://doi.org/10.1111/area.12713

Alaraj, M. M., & Alotaibi, Y. Q. (2019). The Impact of Outside Pressure on Saudi University Students Decision Making. *International Journal of Humanities and Social Science*, *9*(8). https://doi.org/10.30845/ijhss.v9n8p2

Al-Gamal, E., & Long, T. (2013). Psychological distress and perceived support among Jordanian parents living with a child with cerebral palsy: A cross-sectional study. *Scandinavian Journal of Caring Sciences*, *27*(3), 624–631. https://doi.org/10.1111/j.1471-6712.2012.01071.x

Alkan, N. (2014). Humor, Loneliness and Acceptance: Predictors of University Drop-out Intentions. *Procedia - Social and Behavioral Sciences*, *152*, 1079–1086. https://doi.org/10.1016/j.sbspro.2014.09.278

Alsubaie, M. M., Stain, H. J., Webster, L. A. D., & Wadman, R. (2019). The role of sources of social support on depression and quality of life for university students. *International Journal of Adolescence and Youth*, *24*(4), 484–496. https://doi.org/10.1080/02673843.2019.1568887

Andrews, B., & Wilding, J. M. (2004). The relation of depression and anxiety tolife-stress and achievement in students. *British Journal of Psychology*, *95*(509), 521. https://doi.org/https://doi.org/10.1348/0007126042369802

Andrews, G., & Slade, T. (2008). Interpreting scores on the Kessler Psychological Distress Scale (K10). *Technical Physics Letters*, *34*(10), 854–856. https://doi.org/10.1111/j.1467-842X.2001.tb00310.x

Arslan, S., Çardak, M., & Uysal, R. (2013). Student Academic Support as Predictor of Academic Locus of Control in Turkish University Students. *Procedia - Social and Behavioral Sciences*, *106*, 2460–2469. https://doi.org/10.1016/j.sbspro.2013.12.283

Asif, S., Mudassar, A., Shahzad, T. Z., Raouf, M., & Pervaiz, T. (2020). Frequency of depression, anxiety and stress among university students. *Pakistan Journal of Medical Sciences*, *36*(5), 971–976. https://doi.org/10.12669/pjms.36.5.1873

Austin, E. J., Saklofske, D. H., & Mastoras, S. M. (2010). Emotional intelligence, coping and exam-related stress in Canadian undergraduate students. *Australian Journal of Psychology*, *62*(1), 42–50. https://doi.org/10.1080/00049530903312899

Australian Bureau of Statistics. (2012). *Use of the Kessler psychological distress scale in ABS health surveys, Australia, 2007-08*. Cat. No. 4817.0.55.001, ABS, Canberra.

Awang, M. M., Kutty, F. M., & Ahmad, A. R. (2014). Perceived social support and well being: First-year student experience in university. *International Education Studies*, *7*(13), 261–270. https://doi.org/10.5539/ies.v7n13p261

Barg, F. K., Cronholm, P. F., Straton, J. B., Keddem, S., Knott, K., Grater, J., Houts, P., & Palmer, S. C. (2007). Unmet psychosocial needs of pennsylvanians with cancer: 1986-2005. *Cancer*, *110*(3), 631–639. https://doi.org/10.1002/cncr.22820

Barnett, E., & Stamm, L. (2010). *Dual enrollment: A strategy for educational advancement of all students*.

Bautista, L., Relojo, D., Pilao, S. J., Tubon, G., & Andal, M. (2018). Link between lifestyle and self-regulated development as components of academic performance: basis for a psychoeducational intervention. *Journal of Educational Sciences & Psychology*, *8*(1), 68–78.

Beiter, R., Nash, R., McCrady, M., Rhoades, D., Linscomb, M., Clarahan, M., & Sammut, S. (2015). The prevalence and correlates of depression, anxiety, and stress in a sample of college students. *Journal of Affective Disorders*, *173*, 90–96. https://doi.org/10.1016/j.jad.2014.10.054

Bemardon, S., Babb, K. A., Hakim-Larson, J., & Gragg, M. (2011). Loneliness, attachment, and the perception and use of social support in university students. *Canadian Journal of Behavioural Science*, *43*(1), 40–51. https://doi.org/10.1037/a0021199

Berger, J. B., & Braxton, J. M. (1998). Revising Tinto’s interactionalist theory of student departure through theory elaboration: Examining the role of organizational attributes in the persistence process. *Research in Higher Education*, *39*(2), 103–119.

Besser, A., & Zeigler-Hill, V. (2014). Positive Personality Features and Stress among First-year University Students: Implications for Psychological Distress, Functional Impairment, and Self-esteem. In *Self and Identity* (Vol. 13, Issue 1, pp. 24–44). https://doi.org/10.1080/15298868.2012.736690

Böke, B. N., Mills, D. J., Mettler, J., & Heath, N. L. (2019). Stress and coping patterns of university students. *Journal of College Student Development*, *60*(1), 85–103. https://doi.org/10.1353/csd.2019.0005

Boudreault-Bouchard, A. M., Dion, J., Hains, J., Vandermeerschen, J., Laberge, L., & Perron, M. (2013). Impact of parental emotional support and coercive control on adolescents’ self-esteem and psychological distress: Results of a four-year longitudinal study. *Journal of Adolescence*, *36*(4), 695–704. https://doi.org/10.1016/j.adolescence.2013.05.002

Boyes, A., Newell, S., Girgis, A., McElduff, P., & Sanson-Fisher, R. (2006). Does routine assessment and real-time feedback improve cancer patients’ psychosocial well-being? *European Journal of Cancer Care*, *15*(2), 163–171. https://doi.org/10.1111/j.1365-2354.2005.00633.x

Buckley, A. (2014). *The UK engagement survey 2014: The second pilot year*.

Campbell, R., Soenens, B., Beyers, W., & Vansteenkiste, M. (2018). University students’ sleep during an exam period: the role of basic psychological needs and stress. *Motivation and Emotion*, *42*(5), 671–681. https://doi.org/10.1007/s11031-018-9699-x

Cavallo, P., Carpinelli, L., & Savarese, G. (2016). Perceived stress and bruxism in university students. *BMC Research Notes*, *9*(1), 1–6. https://doi.org/10.1186/s13104-016-2311-0

Chan, A., Lomma, C., Chih, H. J., Arto, C., McDonald, F., Patterson, P., Willsher, P., & Reid, C. (2020). Psychosocial consequences in offspring of women with breast cancer. *Psycho-Oncology*, *29*(3), 517–524. https://doi.org/10.1002/pon.5294

Chen, L., Wang, L., Qiu, X. H., Yang, X. X., Qiao, Z. X., Yang, Y. J., & Liang, Y. (2013). Depression among Chinese University Students: Prevalence and Socio-Demographic Correlates. *PLoS ONE*, *8*(3), 1–6. https://doi.org/10.1371/journal.pone.0058379

Chiwuzie, A., & Polytechnic, F. (2021). *Predictors of academic attainment in a Nigerian polytechnic : perceptions of estate management students*  West Africa Built Environment Research (WABER) Conference (793-803) https://www.researchgate.net/publication/353886910_Predictors_of_academic_attainment_in_a_Nigerian_polytechnic_perceptions_of_estate_management_students

Choi, K. H., Park, J. H., Park, J. H., & Park, J. S. (2013). Psychosocial needs of cancer patients and related factors: A multi-center, cross-sectional study in Korea. *Psycho-Oncology*, *22*(5), 1073–1080. https://doi.org/10.1002/pon.3105

Chu-Lien Chao, R. (2012). Managing perceived stress among college students: The roles of social support and dysfunctional coping. *Journal of College Counseling*, *15*(5), 5–22.

Çivitci, A. (2015). The moderating role of positive and negative affect on the relationship between perceived social support and stress in college students. *Kuram ve Uygulamada Egitim Bilimleri*, *15*(3), 565–573. https://doi.org/10.12738/estp.2015.3.2553

Clinciu, A. I. (2013). Adaptation and Stress for the First Year University Students. *Procedia - Social and Behavioral Sciences*, *78*, 718–722. https://doi.org/10.1016/j.sbspro.2013.04.382

Clinton-McHarg, T., Carey, M., Sanson-Fisher, R., D’Este, C., & Shakeshaft, A. (2012). Preliminary development and psychometric evaluation of an unmet needs measure for adolescents and young adults with cancer: The Cancer Needs Questionnaire - Young People (CNQ-YP). *Health and Quality of Life Outcomes*, *10*(1), 13. https://doi.org/10.1186/1477-7525-10-13

Clutterbuck, D., Kochan, F., Lunsford, L., Dominguez, N., Haddock-Millar, J., Lunsford, L. G., Crisp, G., Dolan, E. L., & Wuetherick, B. (2017). Mentoring in Higher Education. *The SAGE Handbook of Mentoring*, 316–332. https://doi.org/10.4135/9781526402011.n20

Coates, H. (2010). Development of the Australasian survey of student engagement (AUSSE). *Higher Education*, *60*(1), 1–17.

Cohen, S., & Wills, T. A. (1985). Stress, social support, and the buffering hypothesis: A theoretical analysis. *Psychological Bulletin*, *98*(2), 310–357.

Crespo, N., Palomo, M. T., & Méndez, M. (2016). *Causes Of Absenteeism In University Students And Its Effects In Causes Of Abseentism In University Students And Its Effects In The Academic Performance*. *November 2012*.

Cummings, E. M., & Kouros, C. D. (2008). Stress and Coping. *Encyclopedia of Infant and Early Childhood Development*, *1*–*3*(3), 267–281. https://doi.org/10.1016/B978-012370877-9.00156-0

Denovan, A., & Macaskill, A. (2013). An interpretative phenomenological analysis of stress and coping in first year undergraduates. *British Educational Research Journal*, *39*(6), 1002–1024. https://doi.org/10.1002/berj.3019

Digdon, N., & Landry, K. (2013). University students’ motives for drinking alcohol are related to evening preference, poor sleep, and ways of coping with stress. In *Biological Rhythm Research, 44*(1), 1-11*.* . https://doi.org/10.1080/09291016.2011.632235

*div-class-title-a-repeated-cross-sectional-survey-assessing-university-students-stress-depression-anxiety-and-suicidality-in-the-early-stages-of-the-covid-19-pandemic-in-poland-div.pdf*. (n.d.).

Dukhan, S. (2020). Value for learning during this time of transformation: the first-year students’ perspective. *Higher Education Research and Development*, *39*(1), 39–52. https://doi.org/10.1080/07294360.2019.1670144

Dyson, G. J., Thompson, K., Palmer, S., Thomas, D. M., & Schofield, P. (2012). The relationship between unmet needs and distress amongst young people with cancer. *Supportive Care in Cancer*, *20*(1), 75–85. https://doi.org/10.1007/s00520-010-1059-7

Ebadi, S., & Rahimi, M. (2019). Mediating EFL learners’ academic writing skills in online dynamic assessment using Google Docs. *Computer Assisted Language Learning*, *32*(5–6), 527–555. https://doi.org/10.1080/09588221.2018.1527362

Eskin, M., Sun, J. M., Abuidhail, J., Yoshimasu, K., Kujan, O., Janghorbani, M., Flood, C., Carta, M. G., Tran, U. S., Mechri, A., Hamdan, M., Poyrazli, S., Aidoudi, K., Bakhshi, S., Harlak, H., Moro, M. F., Nawafleh, H., Phillips, L., Shaheen, A., … Voracek, M. (2016). Suicidal behavior and psychological distress in university students: A 12-nation study. *Archives of Suicide Research*, *20*(3), 369–388. https://doi.org/10.1080/13811118.2015.1054055

Fedorková, J., Nekvapilová, I., & Mikulka, Z. (2020). Study Load at the Beginning of Studies at Military University and Coping Strategies. *International Journal of Education and Information Technologies*, *14*(March 2021), 83–96. https://doi.org/10.46300/9109.2020.14.11

Fedorková, J., Nekvapilová, I., & Mikulka, Z. (2020). Study Load at the Beginning of Studies at Military University and Coping Strategies. *International Journal of Education and Information Technologies*, *14*(March 2021), 83–96. https://doi.org/10.46300/9109.2020.14.11

Field, a P. (2013). Discovering statistics Repeated measures anova. *Discovering Statistics Using SPSS: And Sex and Drugs and Rock ‘n’ Roll (4th Edition)*, *1959*, 1–22.

Flinchbaugh, C., Luth, M. T., & Li, P. (2015). A challenge or a hindrance? Understanding the effects of stressors and thriving on life satisfaction. *International Journal of Stress Management*, *22*(4), 323.

Fyall, R., Stevens, C., Program, H. L., & Manzo, L. (2019). *Understanding Housing and Food Insecurity Among University of Washington Students : An Internal Report*.

Gale, J., & Thalitaya, M. D. eepa. (2015). Mental Health Support Service for University Students. *Psychiatria Danubina*, *27*, S115–S119.

Gallego, J., Aguilar-Parra, J. M., Cangas, A. J., Langer, Á. I., & Mañas, I. (2014). Effect of a mindfulness program on stress, anxiety and depression in university students. *Spanish Journal of Psychology*, *17*(2014), 1–6. https://doi.org/10.1017/sjp.2014.102

Gaultney, J. F. (2010). The prevalence of sleep disorders in college students: Impact on academic performance. *Journal of American College Health*, *59*(2), 91–97. https://doi.org/10.1080/07448481.2010.483708

Gfellner, B. M., & Córdoba, A. I. (2011). Identity Distress, Psychosocial Maturity, and Adaptive Functioning Among University Students. *Identity*, *11*(2), 136–154. https://doi.org/10.1080/15283488.2011.540740

Gide, A. (1967). 済無No Title No Title No Title. *Angewandte Chemie International Edition, 6(11), 951–952.*, *8*(1), 5–24.

Girgis, A., Lambert, S., & Lecathelinais, C. (2011). The supportive care needs survey for partners and caregivers of cancer survivors: Development and psychometric evaluation. *Psycho-Oncology*, *20*(4), 387–393. https://doi.org/10.1002/pon.1740

Gollust, S. E., Eisenberg, D., & Golberstein, E. (2007). Help-Seeking and Access to Mental Health Care in a University Student Population. *Medical Care*, *45*(7), 594–601.

Gomes, A. A., Tavares, J., & De Azevedo, M. H. P. (2011). Sleep and academic performance in undergraduates: A multi-measure, multi-predictor approach. *Chronobiology International*, *28*(9), 786–801. https://doi.org/10.3109/07420528.2011.606518

Granieri, A., Franzoi, I. G., & Chung, M. C. (2021). Editorial: Psychological Distress Among University Students. *Frontiers in Psychology*, *12*, 10–13. https://doi.org/10.3389/fpsyg.2021.647940

Griffith, J. (1984). Emotional support providers and psychological distress among Anglo- and Mexican Americans. *Community Mental Health Journal*, *20*(3), 182–201. https://doi.org/10.1007/BF00808106

Gritsenko, V., Skugarevsky, O., Konstantinov, V., Khamenka, N., Marinova, T., Reznik, A., & Isralowitz, R. (2020). COVID 19 Fear, Stress, Anxiety, and Substance Use Among Russian and Belarusian University Students. *International Journal of Mental Health and Addiction*. https://doi.org/10.1007/s11469-020-00330-z

Gupta, N., Garg, S., & Arora, K. (2016). Pattern of mobile phone usage and its effects on psychological health, sleep, and academic performance in students of a medical university. *National Journal of Physiology, Pharmacy and Pharmacology*, *6*(2), 132–139. https://doi.org/10.5455/njppp.2016.6.0311201599

Hagler, M. A., Christensen, K. M., & Rhodes, J. E. (2021). A Longitudinal Investigation of First-Generation College Students’ Mentoring Relationships During Their Transition to Higher Education. *Journal of College Student Retention: Research, Theory and Practice*. https://doi.org/10.1177/15210251211022741

Hamdan-mansour, A., & Hamdan-Mansour, A. H. M. (2015). Correlates of Resilience Among University Students. *European Psychiatry*, *30*(4), 1945. https://doi.org/10.1016/s0924-9338(15)31485-1

Harris-Reeves, B., & Mahoney, J. (2017). Brief work-integrated learning opportunities and first-year university students’ perceptions of employability and academic performance. *Australian Journal of Career Development*, *26*(1), 32–37. https://doi.org/10.1177/1038416217697974

Hartley, M. T. (2013). Investigating the Relationship of Resilience to Academic Persistence in College Students With Mental Health Issues. *Rehabilitation Counseling Bulletin*, *56*(4), 240–250.

Hautasaari, A., Yamashita, N., & Kudo, T. (2017). Role of CMC in emotional support for depressed foreign students in Japan. *Conference on Human Factors in Computing Systems - Proceedings*, *Part F1276*, 2614–2621. https://doi.org/10.1145/3027063.3053197

Holmes, T. H., & Rahe, R. H. (1967). Schedule of recent experience. *Marriage*, *10*, 50.

Husted, H. S. (2017). The relationship between psychological well-being and successfully transitioning to university. *Undergraduate Thesis*, Scholarship@Western. https://ir.lib.uwo.ca/cgi/viewcontent.cgi?article=1062&context=psychK_uht

Introduction, I. (2006). *University Students ’ Attitudes Toward Online Learning*.

Johnston, N., & Salaz, A. M. (2019). Exploring the Reasons Why University Students Prefer Print over Digital Texts: An Australian Perspective. *Journal of the Australian Library and Information Association*, *68*(2), 126–145. https://doi.org/10.1080/24750158.2019.1587858

Julia, M., & Veni, B. (2012). An analysis of the factors affecting students’ adjustment at a University in Zimbabwe. *International Education Studies*, *5*(6), 244–250. https://doi.org/10.5539/ies.v5n6p244

Kaakinen, P., Suhonen, M., Lutovac, S., & Kaasila, R. (2017). Students experiences of peer-support during a Master’s thesis process. *Clinical Nursing Studies*, *5*(1), 22. https://doi.org/10.5430/cns.v5n1p22

Kabrita, C. S., & Hajjar-Muça, T. A. (2016). Sex-specific sleep patterns among university students in Lebanon: Impact on depression and academic performance. *Nature and Science of Sleep*, *8*, 189–196. https://doi.org/10.2147/NSS.S104383

Kamardeen, I., & Sunindijo, R. Y. (2018). Stressors Impacting the Performance of Graduate Construction Students: Comparison of Domestic and International Students. *Journal of Professional Issues in Engineering Education and Practice*, *144*(4). https://doi.org/10.1061/(ASCE)EI.1943-5541.0000392

Karagiannopoulou, E., & Milienos, F. (2018). Experiences of the Teaching-Learning Environment and Approaches to Learning: Testing the Structure of the “Experiences of Teaching and Learning” Inventory in Relation to Earlier Analyses. *The International Journal of Teaching and Learning in Higher Education*, *30*(3), 506–521.

Kessler, R. C., Andrews, G., Colpe, L. J., Hiripi, E., Mroczek, D. K., Normand, S. L. T., Walters, E. E., & Zaslavsky, A. M. (2002). Short screening scales to monitor population prevalences and trends in non-specific psychological distress. *Psychological Medicine*, *32*(6), 959–976. https://doi.org/10.1017/S0033291702006074

Khan, Z. U. (2018). Attitude of Students Towards Academic Dishonesty in UMT. *SSRN Electronic Journal*, *10*(1), 44–48. https://doi.org/10.2139/ssrn.3270206

Khawaja, N. G., Santos, M. L. R., Habibi, M., & Smith, R. (2013). University students’ depression: A cross-cultural investigation. *Higher Education Research and Development*, *32*(3), 392–406. https://doi.org/10.1080/07294360.2012.697129

Krycak, R. C., Murdock, N. L., & Marszalek, J. M. (2012). Differentiation of Self, Stress, and Emotional Support as Predictors of Psychological Distress. *Contemporary Family Therapy*, *34*(4), 495–515. https://doi.org/10.1007/s10591-012-9207-5

Kuh, G. D. (2001). *The National Survey of Student Engagement: Conceptual framework and overview of psychometric properties*.

Kuittinen, M., & Meriläinen, M. (2011). The effect of study-related burnout on student perceptions. *Journal of International Education in Business*, *4*(1), 42–62. https://doi.org/10.1108/18363261111170586

Lai, C. C. W., & Ma, C. M. S. (2016). The mediating role of social support in the relationship between psychological well-being and health-risk behaviors among Chinese university students. *Health Psychology Open*, *3*(2). https://doi.org/10.1177/2055102916678106

Lawson, H. J., Wellens-Mensah, J. T., & Attah Nantogma, S. (2019). Evaluation of Sleep Patterns and Self-Reported Academic Performance among Medical Students at the University of Ghana School of Medicine and Dentistry. *Sleep Disorders*, *2019*, 1–8. https://doi.org/10.1155/2019/1278579

Leahy, C. M., Peterson, R. F., Wilson, I. G., Newbury, J. W., Tonkin, A. L., & Turnbull, D. (2010). Distress levels and self-reported treatment rates for medicine, law, psychology and mechanical engineering tertiary students: Cross-sectional study. *Australian and New Zealand Journal of Psychiatry*, *44*(7), 608–615. https://doi.org/10.3109/00048671003649052

Lee, W. C., & Matusovich, H. M. (2016). A Model of Co-Curricular Support for Undergraduate Engineering Students. *Journal of Engineering Education*, *105*(3), 406–430. https://doi.org/10.1002/jee.20123

Lefevor, G. T., Sprague, B. M., Boyd-Rogers, C. C., & Smack, A. C. P. (2019). How well do various types of support buffer psychological distress among transgender and gender nonconforming students? In *International Journal of Transgenderism* (Vol. 20, Issue 1, pp. 39–48). https://doi.org/10.1080/15532739.2018.1452172

Lei, X. Y., Xiao, L. M., Liu, Y. N., & Li, Y. M. (2016). Prevalence of Depression among Chinese University Students: A Meta-Analysis. *PLoS ONE*, *11*(4), 1–14. https://doi.org/10.1371/journal.pone.0153454

Lemma, S., Berhane, Y., Worku, A., Gelaye, B., & Williams, M. A. (2014). Good quality sleep is associated with better academic performance among university students in Ethiopia. *Sleep and Breathing*, *18*(2), 257–263. https://doi.org/10.1007/s11325-013-0874-8

Lemma, S., Gelaye, B., Berhane, Y., Worku, A., & Williams, M. A. (2012). Sleep quality and its psychological correlates among university students in Ethiopia: A cross-sectional study. *BMC Psychiatry*, *12*. https://doi.org/10.1186/1471-244X-12-237

Leung, C. H. (2017). University support, adjustment, and mental health in tertiary education students in Hong Kong. *Asia Pacific Education Review*, *18*(1), 115–122. https://doi.org/10.1007/s12564-016-9466-1

Li, C. (2016). Confirmatory factor analysis with ordinal data : Comparing robust maximum likelihood and diagonally weighted least squares. *Behavior Research Methods*, 936–949. https://doi.org/10.3758/s13428-015-0619-7

Lizzio, A. L. F., Wilson, K., & Simons, R. (2010). Studies in Higher Education and Academic Outcomes : University Students ’ Perceptions of the Learning Environment and Academic Outcomes : implications for theory and practice. *Studies in Higher Education*, *5079*(June 2012), 37–41. https://doi.org/10.1080/0307507012009935

López-Rodríguez, M. M., Baldrich-Rodríguez, I., Ruiz-Muelle, A., Cortés-Rodríguez, A. E., Lopezosa-Estepa, T., & Roman, P. (2017). Effects of biodanza on stress, depression, and sleep quality in university students. *Journal of Alternative and Complementary Medicine*, *23*(7), 558–565. https://doi.org/10.1089/acm.2016.0365

Lovell, G. P., Nash, K., Sharman, R., & Lane, B. R. (2015). A cross-sectional investigation of depressive, anxiety, and stress symptoms and health-behavior participation in Australian university students. *Nursing and Health Sciences*, *17*(1), 134–142. https://doi.org/10.1111/nhs.12147

Lund, H. G., Reider, B. D., Whiting, A. B., & Prichard, J. R. (2010). Sleep Patterns and Predictors of Disturbed Sleep in a Large Population of College Students. *Journal of Adolescent Health*, *46*(2), 124–132. https://doi.org/10.1016/j.jadohealth.2009.06.016

Madhyastha, S., Latha, K. S., & Kamath, A. (2014). Stress, Coping and Gender Differences in Third Year Medical Students. *Journal of Health Management*, *16*(2), 315–326. https://doi.org/10.1177/0972063414526124

Martinez, S. M., Esaryk, E. E., Moffat, L., & Ritchie, L. (2021). Redefining Basic Needs for Higher Education: It’s More Than Minimal Food and Housing According to California University Students. *American Journal of Health Promotion*, *35*(6), 818–834. https://doi.org/10.1177/0890117121992295

McDonald, F. E. J., Patterson, P., & Tindle, R. (2020). What young people need when a family member dies of cancer. *Supportive Care in Cancer*, *28*(4), 1631–1638.

McDonald, F. E. J., Patterson, P., White, K. J., Butow, P. N., Costa, D. S. J., & Kerridge, I. (2016). Correlates of unmet needs and psychological distress in adolescent and young adults who have a parent diagnosed with cancer. *Psycho-Oncology*, *25*(4), 447–454. https://doi.org/10.1002/pon.3942

McGaha, V., & Fitzpatrick, J. (2005). Personal and social contributors to dropout risk for undergraduate students. *College Student Journal*, *39*(2), 287–297.

McGillivray, C. J., & Pidgeon, A. M. (2015). Resilience Attributes Among University Students: a Comparative Study of Psychological Distress, Sleep Disturbances and Mindfulness. *European Scientific Journal*, *11*(5), 33–48. http://epublications.bond.edu.au/fsd_papers/221%0Ahttp://eujournal.org/index.php/esj/article/view/5174

McIllmurray, M. B., Francis, B., Harman, J. C., Morris, S. M., Soothill, K., & Thomas, C. (2003). Psychosocial needs in cancer patients related to religious belief. *Palliative Medicine*, *17*(1), 49–54. https://doi.org/10.1191/0269216303pm660oa

McKenzie, K., & Schweitzer, R. (2001). Who succeeds at university? Factors predicting academic performance in first year Australian university students. *Higher Education Research and Development*, *20*(1), 21–33. https://doi.org/10.1080/07924360120043621

McLuckie, A., Matheson, K. M., Landers, A. L., Landine, J., Novick, J., Barrett, T., & Dimitropoulos, G. (2018). The Relationship Between Psychological Distress and Perception of Emotional Support in Medical Students and Residents and Implications for Educational Institutions. *Academic Psychiatry*, *42*(1), 41–47. https://doi.org/10.1007/s40596-017-0800-7

Metzger, I. W., Blevins, C., Calhoun, C. D., Ritchwood, T. D., Gilmore, A. K., Stewart, R., & Bountress, K. E. (2017). An examination of the impact of maladaptive coping on the association between stressor type and alcohol use in college. In *Journal of American College Health* (Vol. 65, Issue 8, pp. 534–541). https://doi.org/10.1080/07448481.2017.1351445

Metzger, I. W., Cooper, S. M., Ritchwood, T. D., Onyeuku, C., & Griffin, C. B. (2017). Profiles of African American College Students’ Alcohol Use and Sexual Behaviors: Associations With Stress, Racial Discrimination, and Social Support. In *Journal of Sex Research* (Vol. 54, Issue 3, pp. 374–385). https://doi.org/10.1080/00224499.2016.1179709

Moore, S. M., Thomas, A. C., Kalé, S., Spence, M., Zlatevska, N., Staiger, P. K., Graffam, J., & Kyrios, M. (2013). Problem Gambling Among International and Domestic University Students in Australia: Who is at Risk? *Journal of Gambling Studies*, *29*(2), 217–230. https://doi.org/10.1007/s10899-012-9309-x

Neves, J. (2018). UK engagement survey. *Sector Results Report Accessible Here: Www. Advance-He. Ac. Uk/Sites/Default/Files/2019-05/Advance_HE_UKES_2018_sector_results_ Report_0. Pdf (Accessed 17 August 2019)*.

Ng, R., Verkooijen, H. M., Ooi, L. L., & Koh, W. P. (2012). Unmet psychosocial needs among cancer patients undergoing ambulatory care in Singapore. *Supportive Care in Cancer*, *20*(5), 1049–1056. https://doi.org/10.1007/s00520-011-1181-1

Nordin, Z. S., Channa, M. A., & Abassi, A. M. (2018). A Quantitative Research for Improving Reading Comprehension of First Year Engineering Students of QUEST, Pakistan Through Metacognitive Strategies. *International Journal of English Linguistics*, *8*(4), 73. https://doi.org/10.5539/ijel.v8n4p73

Nordin, Z. S., Channa, M. A., & Abassi, A. M. (2018). A Quantitative Research for Improving Reading Comprehension of First Year Engineering Students of QUEST, Pakistan Through Metacognitive Strategies. *International Journal of English Linguistics*, *8*(4), 73. https://doi.org/10.5539/ijel.v8n4p73

Oluwole, A., & Oyedun, O. S. (2014). *Psychological Predictors of Post Examination Failure Depression among Preclinical Medical and Dental Students in Ibadan Nigeria*. *4*(6), 216–222. https://doi.org/10.5923/j.ajmms.20140406.04

Omar, S. A. A. A., Khair, A. M. B., Shantakumari, N., Abdelmagied, M., & Hadi, K. M. H. (2020). Perceived sources of stress and stress coping strategies among junior dental students at Ajman university. *Journal of International Dental and Medical Research*, *13*(1), 306–314.

Orzech, K. M., Salafsky, D. B., & Hamilton, L. A. (2011). The state of sleep among college students at a large public University. *Journal of American College Health*, *59*(7), 612–619. https://doi.org/10.1080/07448481.2010.520051

Ozsaker, M., Muslu, G. K., Kahraman, A., Beytut, D., Yardimci, F., & Basbakkal, Z. (2015). A study on the effects of loneliness, depression and perceived social support on problematic internet use among university students. In *Anthropologist* (Vol. 19, Issue 2, pp. 533–542). https://doi.org/10.1080/09720073.2015.11891688

Parnes, M. F., Suárez-Orozco, C., Osei-Twumasi, O., & Schwartz, S. E. O. (2020). Academic Outcomes Among Diverse Community College Students: What Is the Role of Instructor Relationships? *Community College Review*, *48*(3), 277–302. https://doi.org/10.1177/0091552120909908

Patterson, P., & Rangganadhan, A. (2010). Losing a parent to cancer: A preliminary investigation into the needs of adolescents and young adults. *Palliative and Supportive Care*, *8*(3), 255–265. https://doi.org/10.1017/S1478951510000052

Patterson, P., McDonald, F. E. J., Butow, P., White, K. J., Costa, D. S. J., Millar, B., Bell, M. L., Wakefield, C. E., & Cohn, R. J. (2014). Psychometric evaluation of the Sibling Cancer Needs Instrument (SCNI): An instrument to assess the psychosocial unmet needs of young people who are siblings of cancer patients. *Supportive Care in Cancer*, *22*(3), 653–665. https://doi.org/10.1007/s00520-013-2020-3

Patterson, P., McDonald, F. E. J., Butow, P., White, K. J., Costa, D. S. J., Pearce, A., & Bell, M. L. (2013). Psychometric evaluation of the Offspring Cancer Needs Instrument (OCNI): An instrument to assess the psychosocial unmet needs of young people who have a parent with cancer. *Supportive Care in Cancer*, *21*(7), 1927–1938. https://doi.org/10.1007/s00520-013-1749-z

Patterson, P., McDonald, F. E. J., Costa, D. S. J., Tindle, R., Allison, K. R., & Morris, S. E. (2019). Initial validation of a needs instrument for young people bereaved by familial cancer. *Supportive Care in Cancer*. https://doi.org/10.1007/s00520-019-05104-5

Patterson, P., McDonald, F. E. J., Costa, D. S. J., Tindle, R., Allison, K. R., & Morris, S. E. (2020). Initial validation of a needs instrument for young people bereaved by familial cancer. *Supportive Care in Cancer*, *28*(8), 3637–3648.

Patterson, P., McDonald, F. E. J., White, K. J., Walczak, A., & Butow, P. N. (2017). Levels of unmet needs and distress amongst adolescents and young adults (AYAs) impacted by familial cancer. *Psycho-Oncology*, *26*(9), 1285–1292. https://doi.org/10.1002/pon.4421

Patterson, P., Millar, B., & Visser, A. (2011). The Development of an Instrument to Assess the Unmet Needs of Young People Who Have a Sibling with Cancer: Piloting the Sibling Cancer Needs Instrument (SCNI). *Journal of Pediatric Oncology Nursing*, *28*(1), 16–26. https://doi.org/10.1177/1043454210377174

Patterson, P., Pearce, A., & Slawitschka, E. (2011). The initial development of an instrument to assess the psychosocial needs and unmet needs of young people who have a parent with cancer: Piloting the offspring cancer needs instrument (OCNI). *Supportive Care in Cancer*, *19*(8), 1165–1174. https://doi.org/10.1007/s00520-010-0933-7

Peled, Y., & Khaldy, S. (2013). Discrimination, Survival and Tradition as Argumentation for Academic Dishonesty. *Educational Practice and Theory*, *35*(1), 41–61. https://doi.org/10.7459/ept/35.1.04

Peltzer, K., & Pengpid, S. (2015). Nocturnal sleep problems among university students from 26 countries. *Sleep and Breathing*, *19*(2), 499–508. https://doi.org/10.1007/s11325-014-1036-3

Pidgeon, A. M., McGrath, S., Magya, H. B., Stapleton, P., & Lo, B. C. Y. (2014). Psychosocial Moderators of Perceived Stress, Anxiety and Depression in University Students: An International Study. *Open Journal of Social Sciences*, *02*(11), 23–31. https://doi.org/10.4236/jss.2014.211004

Ratelle, C. F., Simard, K., & Guay, F. (2013). University Students’ Subjective Well-being: The Role of Autonomy Support from Parents, Friends, and the Romantic Partner. *Journal of Happiness Studies*, *14*(3), 893–910. https://doi.org/10.1007/s10902-012-9360-4

Robbins, S. B., Le, H., Davis, D., Lauver, K., Langley, R., & Carlstrom, A. (2004). Do Psychosocial and Study Skill Factors Predict College Outcomes? A Meta-Analysis. *Psychological Bulletin*, *130*(2), 261–288. https://doi.org/10.1037/0033-2909.130.2.261

Robotham, D., & Julian, C. (2006). Stress and the higher education student: a critical review of the literature. *Journal of Further and Higher Education*, *30*(2), 107–117. https://doi.org/10.1080/03098770600617513

Rodgers, S., Maloney, B., Ploderer, B., & Brereton, M. (2016). Managing stress, sleep and technologies: An exploratory study of Australian University students. *Proceedings of the 28th Australian Computer-Human Interaction Conference, OzCHI 2016*, 526–530. https://doi.org/10.1145/3010915.3010961

Ruming, K., & Dowling, R. (2017). PhD students’ housing experiences in suburban Sydney, Australia. *Journal of Housing and the Built Environment*, *32*(4), 805–825. https://doi.org/10.1007/s10901-017-9548-3

Ruud, N., Løvseth, L. T., Isaksson Ro, K., & Tyssen, R. (2020). Comparing mental distress and help-seeking among first-year medical students in Norway: Results of two cross-sectional surveys 20 years apart. *BMJ Open*, *10*(8). https://doi.org/10.1136/bmjopen-2020-036968

Sabirova, R., Umurkulova, M., & Kuo, B. C. H. (2020). Academic stress at different yearsof study. *Bulletin of the Karaganda University. Pedagogy Series*, *100*(4), 71–78. https://doi.org/10.31489/2020ped4/71-78

Said, D., Kypri, K., & Bowman, J. (2013). Risk factors for mental disorder among university students in Australia: Findings from a web-based cross-sectional survey. *Social Psychiatry and Psychiatric Epidemiology*, *48*(6), 935–944. https://doi.org/10.1007/s00127-012-0574-x

Salami, S. (2011). Psychosocial Predictors of Adjustment among First Year College of Education Students. *Online Submission*, *8*(2), 239–248.

Sali, R., Roohafza, H., Sadeghi, M., Andalib, E., Shavandi, H., & Sarrafzadegan, N. (2013). Validation of the revised stressful life event questionnaire using a hybrid model of genetic algorithm and artificial neural networks. *Computational and Mathematical Methods in Medicine*, *2013*. https://doi.org/10.1155/2013/601640

Salih, S., Fageehi, M., Hakami, S., Ateya, E., Hakami, M., Hakami, H., Ghazwani, B., Alabdalali, Y., & Mustafa, M. (2021). Academic difficulties among medical students at jazan university: A case–control study. *Advances in Medical Education and Practice*, *12*, 723–729. https://doi.org/10.2147/AMEP.S307554

Sari, W. L., & Fakhruddiana, F. (2019). Internal locus of control, social support and academic procrastination among students in completing the thesis. *International Journal of Evaluation and Research in Education*, *8*(2), 363–368. https://doi.org/10.11591/ijere.v8i2.17043

SARIOĞLU, C. İ. (2020). *Discussions Between Economic Agents: Socio-Economic Studies*. https://orcid.org/0000-0002-1610-8775

Saris, W. E., Satorra, A., & Van der Veld, W. M. (2009). Testing structural equation models or detection of misspecifications? *Structural Equation Modeling*, *16*(4), 561–582.

Satoshi, M. (2021). Who Seeks Social Support from Whom？ Considering Impacts of National and Familial Cultures from Social Ecological Perspectives. *Japanese Journal of Communication Studies*, *49*(2), 79–100. https://doi.org/10.20698/comm.49.2_79

Schlarb, A. A., Claßen, M., Grünwald, J., & Vögele, C. (2017). Sleep disturbances and mental strain in university students: Results from an online survey in Luxembourg and Germany. *International Journal of Mental Health Systems*, *11*(1), 1–10. https://doi.org/10.1186/s13033-017-0131-9

Segrin, C., McNelis, M., & Swiatkowski, P. (2016). Social Support Indirectly Predicts Problem Drinking Through Reduced Psychological Distress. *Substance Use and Misuse*, *51*(5), 608–615. https://doi.org/10.3109/10826084.2015.1126746

SEVİNÇ, S., & GİZİR, C. A. (2014). Factors Negatively Affecting University Adjustment from the Views of First-Year University Students: The Case of Mersin University. *Educational Sciences: Theory & Practice*, *14*(4), 1301–1308. https://doi.org/10.12738/estp.2014.4.2081

Sharp, J., & Theiler, S. (2018). A Review of Psychological Distress Among University Students: Pervasiveness, Implications and Potential Points of Intervention. *International Journal for the Advancement of Counselling*, *40*(3), 193–212. https://doi.org/10.1007/s10447-018-9321-7

Sidelinger, R. J., Frisby, B. N., & Heisler, J. (2016). Students’ out of the classroom communication with instructors and campus services: Exploring social integration and academic involvement. *Learning and Individual Differences*, *47*, 167–171. https://doi.org/10.1016/j.lindif.2016.02.011

Simon, D. (2017). Dual Enrollment and its Impact on College Fershman Persistence: A Modification of Tinto’s Model of Student Departure. *SDSU Doctoral Thesis*.

Soothill, K., Morris, S. M., Harman, J., Francis, B., Thomas, C., & McIllmurray, M. B. (2001). The significant unmet needs of cancer patients: Probing psychosocial concerns. *Supportive Care in Cancer*, *9*(8), 597–605. https://doi.org/10.1007/s005200100278

Stallman, H. M. (2008). Prevalence of psychological distress in university students: Implications for service delivery. *Australian Family Physician*, *37*(8), 673–677.

Stallman, H. M., & Shochet, I. A. N. (2009). *Prevalence of mental health problems in Australian university health services*. *44*(June), 122–127. https://doi.org/10.1080/00050060902733727

Stander, M. (2020). Strategies to help university students avoid plagiarism: a focus on translation as an intervention strategy. *Journal of Further and Higher Education*, *44*(2), 156–169. https://doi.org/10.1080/0309877X.2018.1526260

Stewart, S. M., Betson, C., Lam, T. H., Marshall, I. B., Lee, P. W. H., & Wong, C. M. (1997). Predicting stress in first year medical students: A longitudinal study. *Medical Education*, *31*(3), 163–168. https://doi.org/10.1111/j.1365-2923.1997.tb02560.x

Strnadová, V., Voborník, P., & Provazníková, K. (2020). Stress Load of University Students in Terms of Health Economics. *Proceedings of the International Scientific Conference Hradec Economic Days 2020*, *10*, 740–748. https://doi.org/10.36689/uhk/hed/2020-01-084

Sun, J., Buys, N., Stewart, D., & Shum, D. (2011). Mediating effects of coping, personal belief, and social support on the relationship among stress, depression, and smoking behaviour in university students. *Health Education*, *111*(2), 133–146. https://doi.org/10.1108/09654281111108544

Swash, B., Hulbert-Williams, N., & Bramwell, R. (2014). Unmet psychosocial needs in haematological cancer: A systematic review. *Supportive Care in Cancer*, *22*(4), 1131–1141. https://doi.org/10.1007/s00520-014-2123-5

Talwar, P., Kumaraswamy, N., & Ar, M. F. (n.d.). *Perceived Social Support, Stress and Gender Differences among University Students: A Cross Sectional Study*.

Thurber, C. A., & Walton, E. A. (2012). Homesickness and adjustment in university students. *Journal of American College Health*, *60*(5), 415–419. https://doi.org/10.1080/07448481.2012.673520

Tian, Y., Guo, Z. X., Shi, J. R., Bian, Y. L., Han, P. G., Wang, P., & Gao, F. Q. (2018). Bidirectional Mediating Role of Loneliness in the Association Between Shyness and Generalized Pathological Internet Use in Chinese University Students: A Longitudinal Cross-Lagged Analysis. In *Journal of Psychology: Interdisciplinary and Applied* (Vol. 152, Issue 8, pp. 529–547). https://doi.org/10.1080/00223980.2018.1468309

Tinto, V. (1993). Building community. *Liberal Education*, *79*(4), 16–21.

Tinto, V. (2012). *Completing college: Rethinking institutional action*. University of Chicago Press.

Tsitsas, G., Nanopoulos, P., & Paschali, A. (2019). Life Satisfaction, and Anxiety Levels among University Students. *Creative Education*, *10*(05), 947–961. https://doi.org/10.4236/ce.2019.105071

Universities Australia. (2018). *2017 Universities Australia student finances survey*. *August*.

van Rhijn, T. M., Lero, D., Bridge, K., & Fritz, V. (2016). Unmet Needs: Challenges to Success from the Perspectives of Mature University Students. *Canadian Journal for the Study of Adult Education*, *28*(1), 29–47.

Varathakeyan, A., McDonald, F. E. J., Patterson, P., Nicholson Perry, K., & Allison, K. R. (2018). Accessing support before or after a parent dies from cancer and young people’s current wellbeing. *Supportive Care in Cancer*, *26*(3), 797–805. https://doi.org/10.1007/s00520-017-3891-5

Wada, A., Wagner, D. M., Al Qassab, F., Mohamed, M., Hamad, M., & Al Sharbatti, S. (2016). The relationship between socio-demographic and lifestyle factors and academic performance. *Iranian Journal of Public Health*, *45*(5), 699–701.

Walsh, J. M., Feeney, C., Hussey, J., & Donnellan, C. (2010). Sources of stress and psychological morbidity among undergraduate physiotherapy students. *Physiotherapy*, *96*(3), 206–212. https://doi.org/10.1016/j.physio.2010.01.005

Wibrowski, C. R., Matthews, W. K., & Kitsantas, A. (2017). The Role of a Skills Learning Support Program on First-Generation College Students’ Self-Regulation, Motivation, and Academic Achievement: A Longitudinal Study. *Journal of College Student Retention: Research, Theory and Practice*, *19*(3), 317–332. https://doi.org/10.1177/1521025116629152

Wilcox, P., Winn, S., & Fyvie-Gauld, M. (2005). “It was nothing to do with the university, it was just the people”: The role of social support in the first-year experience of higher education. *Studies in Higher Education*, *30*(6), 707–722. https://doi.org/10.1080/03075070500340036

Williams, S. (2012). The unmet psychosocial and supportive care needs of young adults who have a parent with a non-communicable disease. *PQDT - UK & Ireland*, *October*, 1. https://search.proquest.com/docview/1512406800?accountid=13042%0Ahttp://oxfordsfx.hosted.exlibrisgroup.com/oxford?url_ver=Z39.88-2004&rft_val_fmt=info:ofi/fmt:kev:mtx:dissertation&genre=dissertations+%26+theses&sid=ProQ:ProQuest+Dissertations+%26+Theses+G

Yau, H. K., Sun, H., & Cheng, A. L. F. (2012). Adjusting to university: The Hong Kong experience. *Journal of Higher Education Policy and Management*, *34*(1), 15–27. https://doi.org/10.1080/1360080X.2012.642328
